# Holarctic Species in the *Pluteus romellii* Clade. Five New Species Described and Old Names Reassessed

**DOI:** 10.3390/jof8080773

**Published:** 2022-07-25

**Authors:** Hana Ševčíková, Ekaterina Malysheva, Giuliano Ferisin, Francesco Dovana, Egon Horak, Jacob Kalichman, Oğuzhan Kaygusuz, Renée Lebeuf, Guillermo Muñoz González, Andrew M. Minnis, Stephen D. Russell, Michal Sochor, Bálint Dima, Vladimír Antonín, Alfredo Justo

**Affiliations:** 1 Department of Botany, Moravian Museum, Zelný trh 6, 65937 Brno, Czech Republic; vantonin@mzm.cz; 2 Independent Researcher, 20-75, 194021 St. Petersburg, Russia; ef.malysheva@gmail.com; 3 Associazione Micologica Bassa Friulana, via Vespucci 7, 33052 Cervignano del Friuli, Italy; gferisin@gmail.com; 4 Independent Researcher, 15029 Solero, Italy; francescodovana@gmail.com; 5 Independent Researcher, AT-6020 Innsbruck, Austria; sporax@gmx.net; 6 Department of Ecology and Evolutionary Biology, University of Tennessee, Knoxville, TN 37996, USA; jkmycetes@gmail.com; 7 Department of Plant and Animal Production, Atabey Vocational School, Isparta University of Applied Sciences, Isparta 32670, Turkey; okaygusuz03@gmail.com; 8 Renée Lebeuf, 775 Rapide Nord, Saint-Casimir, QC G0A 3L0, Canada; renee.lebeuf@gmail.com; 9 Guillermo Muñoz González, C/Tudela, 20, 50650 Gallur, Spain; guillermomunoz1981@gmail.com; 10 Andrew Minnis, USDA-APHIS-PPQ, Seattle Plant Inspection Station 835 S. 192nd St., Suite 1600, SeaTac, WA 98148, USA; minnisncf@gmail.com; 11 The Hoosier Mushroom Society, 3912 S Carey St, Marion, IN 46953, USA; steve@hoosiermushrooms.org; 12 Centre of the Region Haná for Biotechnological and Agricultural Research, Crop Research Institute, Šlechtitelů 29, 78371 Olomouc, Czech Republic; michal.sochor@volny.cz; 13 Department of Plant Anatomy, Institute of Biology, Eötvös Loránd University, Pázmány Péter sétány 1/C, H-1117 Budapest, Hungary; cortinarius1@gmail.com; 14 New Brunswick Museum, 277 Douglas Ave., Saint John, NB E2K 1E5, Canada

**Keywords:** Agaricales, Pluteaceae, phylogeny, sect. *Celluloderma*, taxonomy

## Abstract

We studied the taxonomy of *Pluteus romellii*, and morphologically similar Holarctic species in the/romellii clade of section *Celluloderma*, using morphological and molecular data (nrITS, *TEF1-α*). *Pluteus romellii* is lectotypified and epitypified and accepted as an exclusively Eurasian species. *Pluteus lutescens* and *P. pallescens* are considered synonyms of *P. romellii*. *Pluteus fulvibadius* is accepted as a related, but separate, North American species. Five species in the/romellii clade are described as new to science: two from North America (*P. austrofulvus* and *P. parvisporus*), one from Asia (*P. parvicarpus*), one from Europe (*P. siccus*), and one widely distributed across the Holarctic region (*P. vellingae*). Basidioma size, pileus color, lamellae color, basidiospore size, hymenial cystidia shape and size, habitat and geographical distribution help separate the species described here, but in some instances only molecular data allows for confident identification. The current status of *P. californicus*, *P. melleipes*, *P. romellii* var. *luteoalbus*, *P. splendidus*, *P. sternbergii* and *P. sulphureus* is discussed.

## 1. Introduction

*Pluteus* Fr. is a genus of saprotrophic mushroom-forming fungi belonging to the family *Pluteaceae* Kotl. & Pouzar [1]. Within *Pluteaceae*, the genus *Pluteus* is easily recognizable from related genera *Volvariella* Speg. and *Volvopluteus* Vizzini, Contu & Justo by lacking a volva, but some species historically classified in the genus *Chamaeota* (W.G. Sm.) Earle possess partial veil [2], represented in the form of an annulus. These genera are also distinguishable by basidiospore size and pileipellis structure [2]. The genus *Pluteus* is characterized morphologically by the combination of basidiomata with free lamellae, a pinkish spore print, inverse hymenophoral trama, smooth, globose to oblong spores, and the presence of cheilocystidia and often also pleurocystidia [3,4]. This genus currently includes about five hundred accepted species [5], but a lot of them need taxonomic revision using modern phylogenetic methods. *Pluteus* species are widely distributed in Holarctic [6,7,8,9,10,11,12,13,14,15,16,17,18,19,20,21,22,23,24,25,26,27,28,29,30], Neotropical [31,32,33,34,35,36,37,38], Paleotropical [39,40,41,42,43,44,45,46] and Austral ecosystems [47,48,49]. The genus has traditionally been subdivided into morphological groups (sections, subsections) according to the characteristics of the hymenial cystidia and the type of pileipellis [3,22,23,32]. Molecular phylogenetic work [2,50] supports three sections (*Pluteus*, *Hispidoderma* Fayod and *Celluloderma* Fayod). *Pluteus* section *Celluloderma* accommodates species with non-metuloid cystidia and pileipellis as an epithelium or hymeniderm with clavate or spheropedunculate elements (avQ ≤ 3). Some species with non-metuloid cystidia and pileipellis as a cutis (e.g., *P. ephebeus* (Fr.) Gillet and related taxa) belong phylogenetically in sect. *Celluloderma* but are quite different, morphologically, from the majority of the species in the section [50]. The traditional subdivision of the sect. *Celluloderma* in subsections *Mixtini* Singer (with additional elongated elements in the pileipellis) and *Eucellulodermini* Singer (without them) is not supported by molecular data, as species with elongated elements occur throughout the sect. *Celluloderma* [50,51].

In modern European taxonomic literature, *Pluteus romellii* is commonly accepted as the correct name for a species in sect. *Celluloderma* with a brown pileus, bright yellow colors on the stipe and a pileipellis without elongated elements [4,50,52]. Most authors have accepted the name *Pluteus lutescens* (Fr.) Bres. as a younger taxonomic synonym of *P. romellii* e.g., [4,22,23,26,53]. *Pluteus romellii* has been cited throughout Europe e.g., [4,22,52,54,55,56,57], and has also been reported from Japan [50,58].

In North America, different names have been used for collections resembling the European concept of *Pluteus romellii*. Homola [21] made the first major taxonomic revision focused on sect. *Celluloderma* in the USA. In that work, the following species in subsection *Eucellulodermini* (as “subsection *Celluloderma*”) with yellow-green to brown-colored pilei and a yellow stipe were accepted: *P. rugosidiscus* Murrill, *P. californicus* McClatchie, *P. lutescens* (Fr.) Bres. and *Pluteus melleipes* Murrill. Furthermore, two varieties were distinguished in *P. lutescens*: var. *lutescens* and a second, unnamed variety (“var. sp.”) for collections made in Western North America. Homola [21] also accepted *Pluteus fulvibadius* Murrill as a species with a yellow stipe, but he described that taxon as having elongated elements in the pileipellis, therefore placing it in subsect. *Mixtini*. Minnis & Sundberg [27] published the most recent monograph of sect. *Celluloderma* in the USA and adopted a different taxonomic arrangement of those species than Homola. Based on the study of numerous collections, as well as type specimens, Minnis & Sundberg accepted *Pluteus fulvibadius* Murrill as the correct name for the North American *romellii*-like collections in subsect. *Eucellulodermini*, disputing the placement of the species in subsect. *Mixtini* from Homola [21]. For the other yellow-stipe species accepted by Homola, Minnis & Sundberg [27] placed *P. melleipes* in synonymy with *P. fulvibadius*; rejected the characterization of *P. californicus* as a species with a yellow stipe; and placed *P. rugosidiscus* as a synonym of *P. chrysophlebius* (Berk. & Ravenel) Sacc.

The first phylogenetic data presented by Justo et al. [2,50] clarified some of the previous taxonomic problems, but also left some questions open for future research. DNA data (nrITS) showed that *P. rugosidiscus* and *P. chrysophlebius* are separate species, and that both species are unrelated to the/romellii clade. A total of seven collections assignable to *Pluteus romellii* from Europe, Asia and North America were included in that analysis, and they were recovered as one relatively well-supported clade, sister to *P. aurantiorugosus*. Those two species, together with some South American taxa, formed a well-supported lineage within sect. *Celluloderma*, recognized as the “/romellii-aurantiorugosus clade”. While the nrITS sequences from different geographical origins were not identical to each other, no particular phylogeographic structure within *P. romellii* was evident in the phylogenetic analyses presented [2]. Other species of sect. *Celluloderma* analyzed in the same work did show clear phylogeographic structure (e.g., *P. phlebophorus*, *P. aurantiorugosus*, *P. chrysophlebius*). An additional sequence identified as *P. romellii* in GenBank (AY854065) was included in the analyses, and it was clear that it represented a different species from all other collections identified as *P. romellii*, but no further clarifications about its true identity could be made at that point [2]. The phylogenies of Menolli et al. [39], while focused on South American taxa, also presented a broader sampling of *P. romellii* collections from North America, and some internal phylogenetic structure of the/romellii clade can be observed in the nrITS tree presented by these authors. The broader clade including *P. romellii*, *P. aurantiorugosus* and related taxa was referred to as “/aurantiorugosus clade” in that article.

In the present study, we focus on the taxonomy and biogeography of *Pluteus romellii* and phylogenetically related species present in the temperate and boreal areas of the northern Hemisphere, which approximately corresponds to the Holarctic region [59]. We aim to clarify the status of *P. romellii* and related taxa mentioned above, including the identity of some additional very poorly known species such as *Pluteus sternbergii* Velen. [15], *P. sulphureus* Velen. [15] and *P. splendidus* A. Pearson [60]. Based on the morphological and molecular variation (nrITS and/or *TEF1-α*) of 117 collections we: (i) define the distribution limits of *P. romellii* sensu stricto as an exclusively Eurasian species. *Pluteus pallescens* P.D. Orton is considered a synonym of *P. romellii*; (ii) recognize four additional species in the *P. romellii* species complex: three exclusively North American species (*P. fulvibadius*, *P. austrofulvus* sp. nov., *P. parvisporus* sp. nov.) and one additional Eurasian species (*Pluteus* aff. *romellii*) not formally described here; and (iii) recovered a lineage in the/romellii clade, separate from the *P. romellii* species complex, with three additional species described here as new to science (*P. parvicarpus* sp. nov., *P. siccus* sp. nov., *P. vellingae* sp. nov.).

## 2. Materials and Methods

### 2.1. Morphology

Macroscopic descriptions of newly collected specimens are based on fresh basidiomata, and microscopic descriptions are based on dried specimens; except for the holotypes of *P. sternbergii* and *P. sulphureus* which were preserved in Velenovský’s solution [61]. Color abbreviations follow RAL Design color range system (https://www.ralcolorchart.com/ral-design; accessed on 3 April 2022) or Munsell Soil-Color charts [62]; herbarium abbreviations are those according to [63]. Microscopic features were described from dried material mounted in 10% KOH and Congo Red with a magnification of 500×, 600× and 1000×. Terminology follows [24]. Abbreviations: avl = average length, avw = average width, Q = quotient of length and width, avQ = average Q. Average values for each species are given as intervals of the individual average values for each collection examined. The notation [X, Y, Z] indicates that measurements were made on X basidiospores, in Y basidiomata from Z collections. The newly collected specimens are deposited in the herbaria BRNM, LE, NBM, PUL and the personal herbaria of GF, OK and GM. All holotypes are stored in public herbaria. MycoBank numbers were used as unique identifiers for each new species.

### 2.2. Molecular Phylogeny

#### 2.2.1. DNA Extraction, Amplification, Sequencing and Sequence Alignment

For DNA extraction, small fragments of dried basidiomata were used. Sequencing of the collections deposited in the herbaria BRNM, PRM and P was performed by M. Sochor, following the molecular methods described by Ševčíková et al. [64]. The type material of *P. pallescens*, deposited in K, was studied by B. Dima, and the molecular methods followed [65,66]. The total genomic DNA of OK collections was extracted in accordance with Kaygusuz et al. [67]. For collections in LE, the DNA extraction procedure completely corresponded to the manufacturing protocol of the Phytosorb Kit (ZAO Syntol). New molecular sequences for collections at NBM were generated at ALVALAB (http://www.alvalab.es/index.html. Last accessed 1 July 2022).

The following primers were used for amplification and sequencing: ITS1F-ITS4/ITS4B [68,69] for the internal transcribed spacer (nrITS: nrITS1-5.8S-nrITS2) fragment; EF1-983F and EF1-1567R for part of the translation elongation factor 1-alpha (*TEF1-α*) [70]. PCR (Polymerase Chain Reaction) products were purified applying the GeneJET Gel Extraction Kit (Thermo Fisher Scientific, Waltham, MA, USA) or by precipitation with polyethylene glycol (10% PEG 6000 and 1.25 M NaCl in the precipitation mixture). Sequencing (Sanger method) was carried out at the institutions of the authors, at Macrogen Europe (Amsterdam, The Netherlands) and at ALVALAB.

#### 2.2.2. Phylogenetic Analyses

Raw data were edited and assembled in MEGA 10 [71], with the CodonCode Aligner package (CodonCode Corp., Centerville, MA, USA) and ChromasPro (Technelysium). We assembled a nrITS dataset of all available sequences phylogenetically close to *P. romellii* (“/romellii-aurantiorugosus clade” in [2], “/aurantiorugosus clade” in [39]. This includes 57 newly generated nrITS sequences for this study, and 56 sequences generated in previous studies or available in public databases and biodiversity repositories (GenBank, UNITE, BOLD, iNaturalist). A total of 113 nrITS sequences were used in the final datasets, including voucher-based and environmental sequences. We assembled a *TEF1-α* dataset of 40 sequences, 35 of them newly generated for this study. In all datasets, we included *P. phlebophorus* and *P. rugosidiscus* as outgroup taxa, based on previous phylogenetic work on *Pluteus* [2,40,50]. All sequences used in the analyses are listed in Table S1. Sequences were aligned using MAFFT version 7 [72] and the strategy FFT-NS-i. The alignment was inspected and manually corrected in AliView [73]. No topological conflicts were detected in the phylogenetic analyses of the nrITS and *TEF1-α* datasets (detailed below), so a combined dataset was created by concatenating the nrITS and *TEF1-α* matrices.

For all three datasets (nrITS, *TEF1-α* and nrITS + *TEF1-α*), two separate phylogenetic analyses were run: (i) maximum likelihood (ML) analyses using RAxML 8.2.10 under a GTRGAMMAI model as recommended [74], with 100 rapid bootstrap (BS) replicates; (ii) Bayesian inference (BI) analyses using MrBayes 3.2.7 [75] for 10 million generations under a GTRGAMMAI model with four chains, and trees sampled every 1000 generations. The initial burn-in phase was set to 2.5 million generations, and this value was confirmed to be adequate by checking the graphic representation of the likelihood scores of the sampled trees. Additionally, we also confirmed that the standard deviation of split frequencies was <0.05, and that potential scale reduction factor (PRSF) values were close to 1, as detailed in Ronquist et al. [76]. All analyses were run using resources at the CIPRES Science Gateway [77]. All phylogenetic trees were initially visualized using FigTree (http://tree.bio.ed.ac.uk/software/figtree/. Last Accessed 1 July 2022). Trees were exported from FigTree as SVG files and edited in Adobe Illustrator for final presentation.

## 3. Results

### 3.1. Phylogeny

The nrITS dataset comprises 113 sequences and 760 characters (gaps included). The *TEF1-α* dataset comprises 40 sequences and 855 characters (gaps included). The combined nrITS + *TEF1-α* dataset consisted of 115 combined sequences and a total of 1615 characters (gaps included). There were no major differences in the overall topologies of the best tree from the ML analysis and the consensus tree from the BI analysis for any of the datasets.

**Figure 1:**
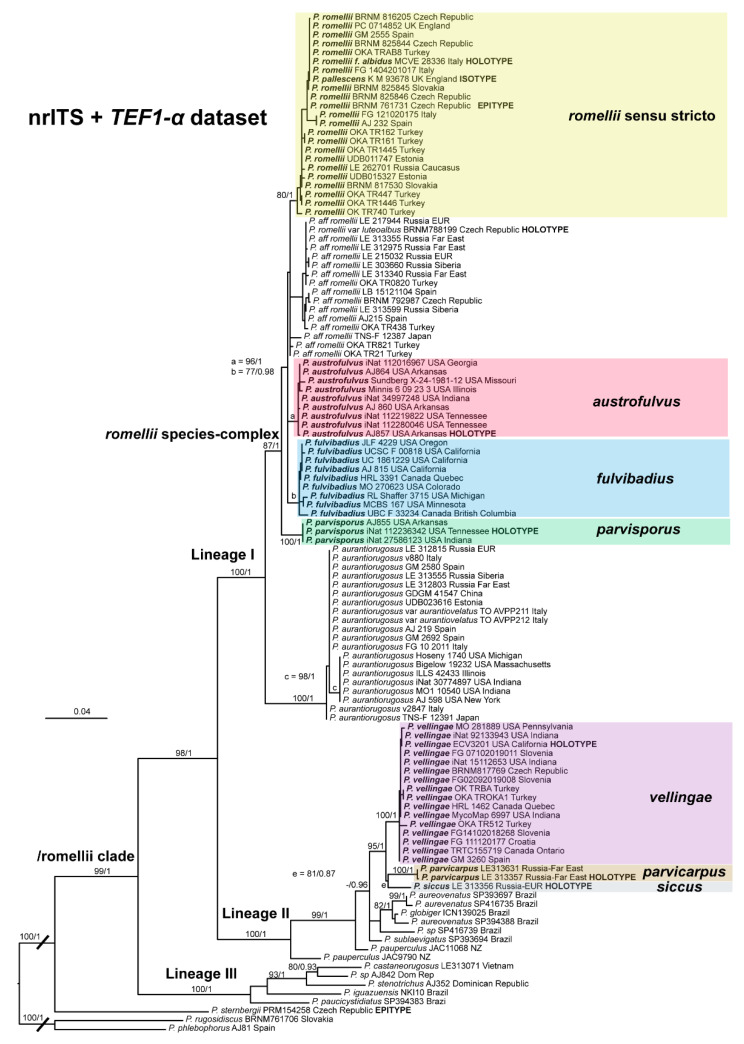
Best tree from the ML analysis of the nrITS + *TEF1-α* dataset. BS values (≥70%) and PP values (≥0.90) are shown on or below the branches. Root length has been reduced to accommodate graphical representation. Scale bar indicates the mean number of nucleotide substitutions per site.

**Figure 2:**
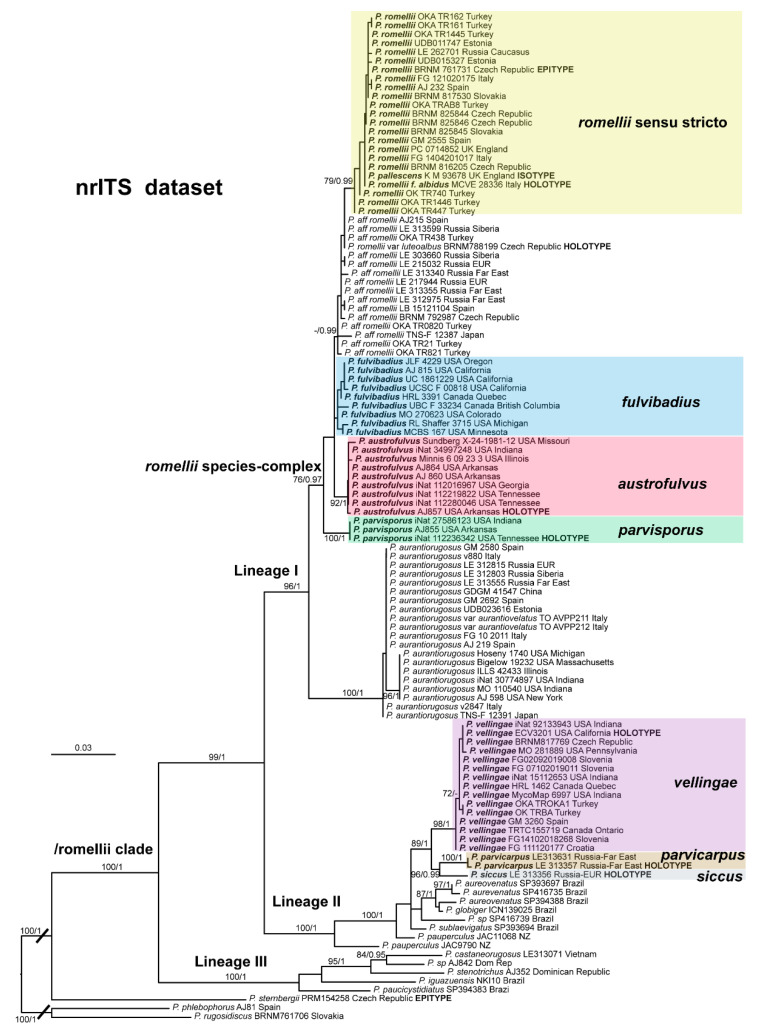
Best tree from the ML analysis of the nrITS dataset. BS values (≥70%) and PP values (≥0.90) are shown on or below the branches. Root length has been reduced to accommodate graphical representation. Scale bar indicates the mean number of nucleotide substitutions per site.

**Figure 3:**
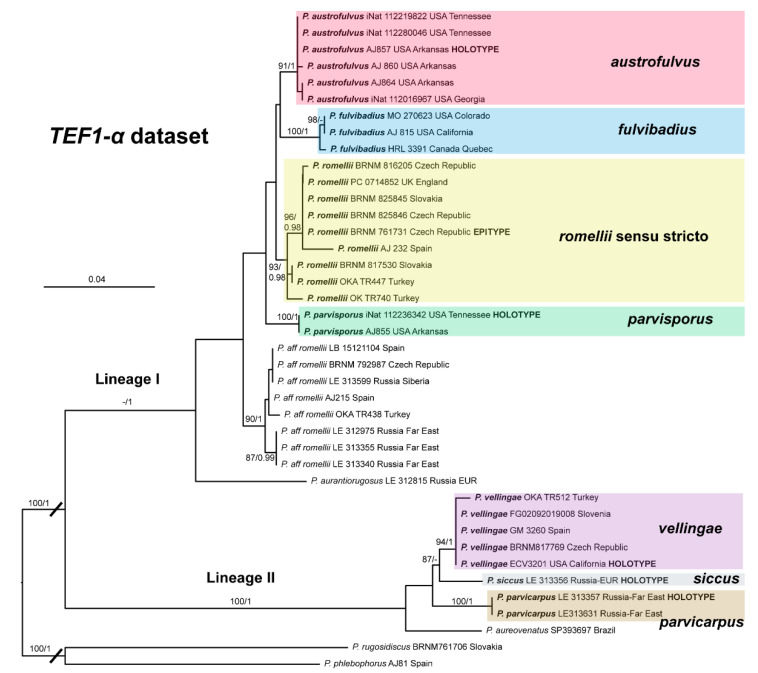
Best tree from the ML analysis of the *TEF1-α* dataset. BS values (≥70%) and PP values (≥0.90) are shown on or below the branches. Root length has been reduced to accommodate graphical representation. Scale bar indicates the mean number of nucleotide substitutions per site.

In Figure 1, we present the best tree from the ML analysis of the nrITS + *TEF1-α* dataset, which will be the main reference point for the taxonomic discussion. The individual nrITS and *TEF1-α* trees (ML analyses) are presented in Figure 2 and Figure 3, respectively, as some aspects of these single-gene phylogenies are relevant to the taxonomic discussions.

For clarity, throughout the paper, the term “/romellii clade” is used for the clade previously recognized as “/romellii-aurantiorugosus clade” [2] and “aurantiorugosus clade” [39]. This clade always appears as a distinct and well-supported lineage in broader phylogenetic analyses of sect. *Celluloderma* (data not shown). The term “*romellii* species complex” is reserved for the least inclusive clade that includes *P. romellii* sensu stricto and closely related species, but not *P. aurantiorugosus*. Thus, some of the species discussed here are part of both the/romellii clade and the *romellii* species complex, while others are part of one but not the other.

A clade or relation is considered to be strongly supported if it receives BS ≥ 90% and posterior probability/probabilities (PP) = 1, and well supported if it receives BS ≥ 70% and PP ≥ 0.95.

Within the/romellii clade, three strongly supported lineages are recovered in the combined nrITS + *TEF1-α* dataset:

(i). Lineage I: Includes the *romellii* species complex (*P. romellii*, *P.* aff. *romellii*, *P. austrofulvus*, *P. fulvibadius*, *P. parvisporus*) and *P. aurantiorugosus*. All taxa in the *romellii* species complex are strongly or well supported, with the exception of *Pluteus* aff. *romellii*. None of the sister-taxa relationships within the *romellii* species complex receive significant support.
(ii). Lineage II: Includes a strongly supported clade with three of the species described here as new (*P. vellingae*, *P. parvicarpus*, *P. siccus*) and sequences identified as several Southern Hemisphere taxa (*P. aureovenatus*, *P. globiger*, *P. sublaevigatus*, *P. pauperculus*).
(iii). Lineage III: Includes the recently described *P. castaneorugosus* (from Vietnam) and several neotropical taxa (*P. stenotrichus*, *P. iguazuensis*, *P. paucicystidiatus*).

*Pluteus sternbergii* appears as sister to the/romellii clade.

The topology of the nrITS tree is shown in Figure 2. The overall topology is very similar to the one of the combined datasets, but support values for some of the taxa and relations discussed here are slightly or significantly lower (e.g., *P. fulvibadius*).

The topology of the *TEF1-α* tree is shown in Figure 3. All species in the *romellii* species complex are recovered as separate from each other in strongly or well-supported clades. The relations between these species are slightly different from the ones recovered in the nrITS and combined datasets, but none of these relations receive any significant support. In the *TEF1-α*, tree *Pluteus siccus* appears as sister to *P. vellingae*, which is different from the topology recovered in the nrITS and combined datasets, where *P. siccus* is sister to *P. parvicarpus*. The position of *P. siccus* in the nrITS tree is strongly supported (96%/0.99) while in the *TEF1-α* tree it is not (87% and no support in Bayesian tree).

### 3.2. Taxonomy

Below, we present the detailed descriptions of *Pluteus romellii* and *P. fulvibadius*, as accepted and delimited here. We propose the following Holarctic species in the/romellii clade as new: *P. austrofulvus* (Eastern Nearctic), *P. parvicarpus* (Eastern Palearctic), *P. parvisporus* (Eastern Nearctic), *P. siccus* (Eastern Palearctic) and *P. vellingae* (Holarctic). We establish the synonymy of *P. pallescens* with *P. romellii*, accept the previously suggested synonymy of *P. lutescens* and *P. romellii*, and discuss the current status of several older names related to the taxa in the/romellii clade.

**Figure 4:**
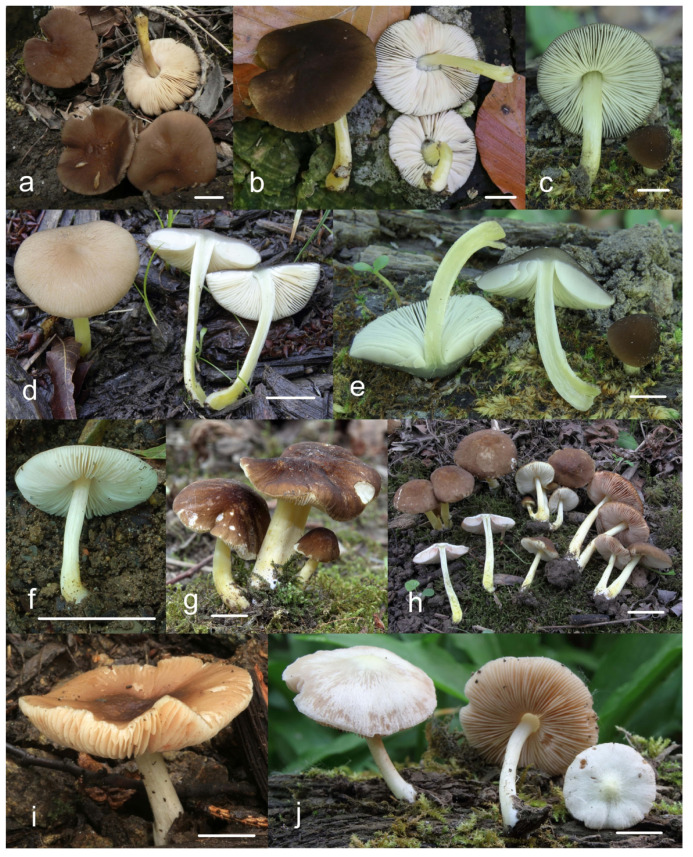
*Pluteusromellii* basidiomata in situ: (**a**). epitype BRNM 761731, (**b**). OKA-TR1445, (**c**,**e**). FG27052020, (**d**). FG24052020, (**f**). NBM-F-009322, (**g**,**h**). FG 14042019017, (**i**). BRNM 781260, (**j**). *Pluteus* *romellii* f. *albidus* FG 25042018003. Scale bars 1 cm.

**Figure 5:**
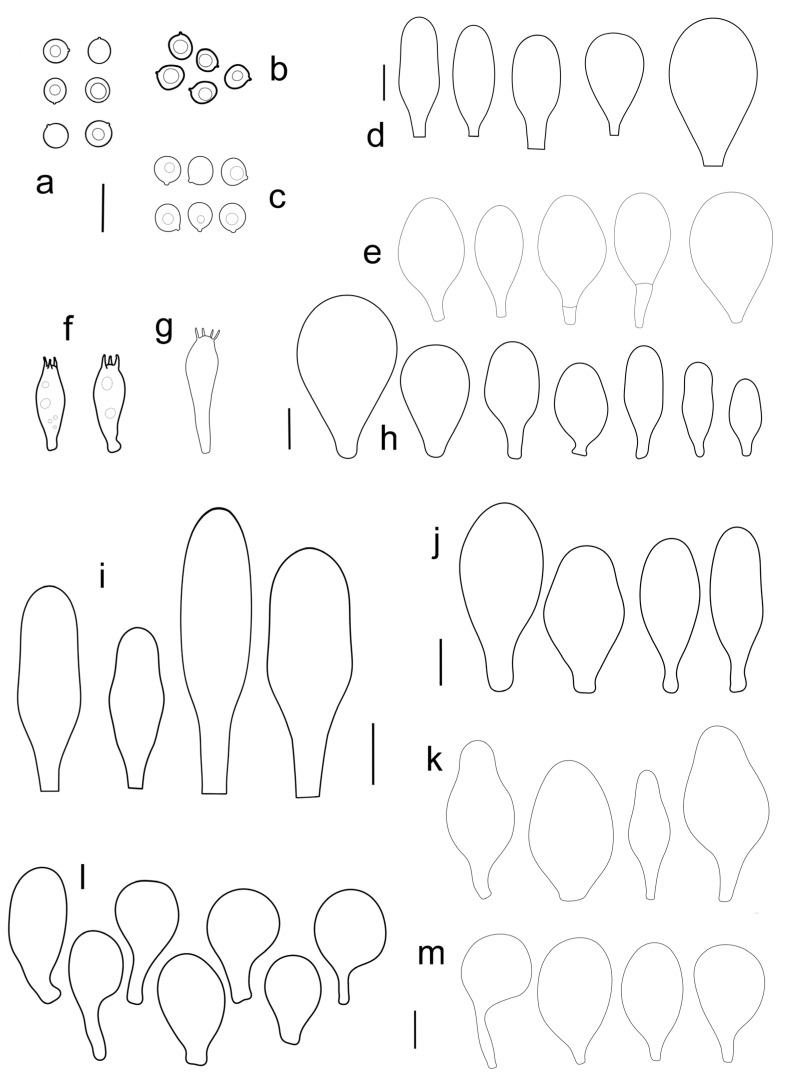
*Pluteusromellii* microcharacters: (**a**–**c**). basidiospores, (**a**). epitype BRNM 761731, (**b**). FG 18029 (**c**). LE 262701; (**d**). BRNM 761731 cheilocystidia; (**e**). LE 262701 cheilocystidia; (**f**). FG 18029 basidia; (**g**). LE 262701 basidium; (**h**). FG 18029 cheilocystidia; (**i**–**k**). pleurocystidia (**i**). BRNM 761731, (**j**). FG 18029, (**k**). LE 262701; (**l**,**m**). pileipellis elements (**l**). FG 18029, (**m**). LE 262701. Scale bars 10 μm.

***Pluteus romellii*** (Britzelm.) Lapl., Dictionnaire Iconographique des Champignons Supérieurs (Hyménomycètes): 533 (1894). Figure 4 and Figure 5.

Basionym: *Agaricus romellii* Britzelm., Hymenomyceten aus Südbayern VIII: 5 (1891).

Lectotype: MBT10008068, designated here. Illustration of *Agaricus romellii* in Britzelmayr, Hymenomyceten aus Südbayern VIII: F. 113 Hyporhodii (1891).

Epitype: MBT10008039, designated here. Europe: CZECH REPUBLIC: Lanžhot, Cahnov National Nature Reserve, on fallen oak trunk spanning the river and on soil near that trunk, 11 April 2014, leg. H. Ševčíková (BRNM 761731).

Syn.: *Agaricus nanus* var. *lutescens* Fr., Epicr. syst. mycol. (Upsaliae): 141 (1838) (1836–1838)

*Pluteus lutescens* (Fr.) Bres., Icon. Mycol. (Paris) 11: 544 (1929).

*Pluteus nanus* subsp. *lutescens* (Fr.) Konrad & Maubl., Icon. Select. Fung. 6: 55 (1930).

*Pluteus nanus* var. *lutescens* (Fr.) P. Karst., Bidr. Känn. Finl. Nat. Folk 32: 256 (1879).

*Pluteus pallescens* P.D. Orton, Trans. Br. mycol. Soc. 43(2): 360 (1960).

Isotype: Great Britain, Norfolk, Surlingham, Bossington, Wheatfen Carr, 7 July 1958 leg. P.D. Orton (K(M)93678).

*Pluteus satur* f. *pallescens* (P.D. Orton) Citérin & Eyssart., Doc. Mycol. 28 (no. 111): 56 (1998).

Misapplied name: *Pluteus chrysophaeus* sensu Métrod in Rev. Mycol. 7: 19 (1943).

Pileus 12–60(–80) mm broad, hemispherical or campanulate-convex, rarely conical, later plano-convex to applanate, without or with obtuse umbo; hygrophanous or not; translucently striate to sulcate at the margin or not, sometimes slightly eroded; usually yellow-brown, light brown to dark brown, rarely cinnamon brown, reddish brown or beige; with darker brown, rarely almost blackish center; surface smooth, rarely indistinctly velvety, matte or not, strongly to indistinctly radially venose from the center towards the margin. Lamellae L = 24–90(–110), l = 1–3, free, moderately crowded to crowded, slightly to distinctly ventricose, 2–10 mm broad, whitish or yellowish when young, later pink, often with yellow tinges, with white or concolorous, rarely yellowish flocculose even or eroded edge. Stipe (22–)25–91 × 1.5–7(–9) mm, cylindrical, sometimes with slightly broadened base, solid or fistulose; surface smooth or longitudinally innately fibrillose, covered with concolorous floccules especially at the lower part, whitish to yellow, rarely with very pale brownish tinge, with more distinct color at the base. Basal mycelium tomentose or absent, whitish to very pale yellowish when present. Context watery, white in the pileus, pale yellow, yellow to lemon-yellow in the stipe, sometimes whitish only with yellow tinge at the base or with very pale brownish tinge. Smell indistinct or slightly sweet, taste indistinct or mild.

Basidiospores [600, 14, 13] (5.1–)5.4–8.4(–8.8) × (4.0–)4.5–6.6(–7.6) µm, avl × avw = 6.5–7.3 × 5.3–6.1 μm, Q = (1.0–)1.09–1.36(–1.41), avQ = 1.16–1.24; broadly ellipsoid or subglobose, rarely ellipsoid, some ovoid or globose, thick-walled. Basidia (18–)22–34(–39) × 7–11 µm, subcylindrical or subclavate, 4-spored. Pleurocystidia scarce to numerous, (32–)40–85(–90) × (13–)18–34(–48) µm, broadly clavate to clavate, broadly cylindrical to utriform or subutriform, rarely spathulate or subfusiform with long neck, thin-walled, hyaline. Lamellar edge sterile, cheilocystidia crowded, (20–)27–60(–80) × (7–)10–34(–44) µm, variable in shape, narrowly to broadly clavate, clavate with median constriction, broadly cylindrical to cylindrical, narrowly to broadly utriform, subutriform, thin-walled, hyaline or with slightly refractive intracellular pigment. Pileipellis an euhymeniderm of spheropedunculate, subglobose, broadly clavate and rare narrowly clavate to almost cylindrical elements (30–)33–60(–80) × (10–)20–40(–50) µm, with yellow-brown or light brown, at the center brown to dark brown, rarely very pale brownish intracellular pigment, thin-walled to slightly thick-walled. Stipitipellis a cutis, hyphae 5–12 µm wide, hyaline to yellow, thin to slightly thick-walled. Caulocystidia absent or present only sporadically on the upper part of the stipe, colorless or with pale yellow-brown content, similar to cheilocystidia. Clamp connections absent in all studied tissues. 

Habit, habitat, phenology and distribution: solitary or in groups, growing on soil, on decayed wood or twigs of broadleaved trees, fruiting from March to October, rarely November. Europe and Western Asia (Turkey).

**Additional collections examined**: Europe. CZECH REPUBLIC: Popůvky near Brno, aluvium of Augšperský stream, decayed twigs of deciduous trees, 29 May 2019, leg. H. Ševčíková (BRNM 816205); Kuřim–Bělč, Bělčský stream valley, on soil under *Alnus*, *Salix*, *Tilia*, *Corylus* and *Acer*, 27 September 2018, leg. V. Antonín, H. Ševčíková (BRNM 825844 originally as *P. pallescens*); Brno–Bystrc, Jelení žlíbek Nature Reserve, on fallen trunk of *Tilia*, 17 June 2016, leg. H. Ševčíková (BRNM 781175, originally as *P. pallescens*); ca. 300 m south of the Jelení žlíbek, on a forest path among wood remains, 1 July 2016, leg. V. Antonín, H. Ševčíková (BRNM 781260, originally as *P. pallescens*). GREAT BRITAIN: Oxfordshire, Woodstock, Blenheim park, in grass under *Tilia* sp., 4 September 1965, leg. D.A. Reid, det. P.M. Reid (PC 0714852, originally as *Pluteus lutescens*). ITALY: Farra d’Isonzo, Isonzo (Soca) river, 12 October 2017, leg. G. Ferisin, FG 18029; *ibidem*, 14 April 2019, leg. G. Ferisin, FG 14042019017. RUSSIA: Caucasus, Karachayevo-Circassian Republic, Teberda Biosphere Reserve, Arkhyzzone, bank of Kizgich river, mixed forest (*Abies*, *Fagus*, *Populus*), on wood of deciduous tree, 21 August 2009, leg. E.F. Malysheva (LE 262701). SLOVAKIA: Hybe, Hybická tiesňava Nature Monument, decayed twig of *Corylus*, 21 August 2019, leg. V. Halasů (BRNM 817530); Ružomberok, Černovské lúky, on soil under *Corylus*, *Acer campestre*, *Crataegus*, 22 August 2019, leg. V. Halasů (BRNM 825845). SPAIN: Asturias, Vigidel, on decaying wood of deciduous tree, 4 October 2005, leg. P. Siquier, L. Parra, *AJ 232* (NBM-F-009321). Navarra, Bertizarana, on the edge of a road, under *Quercus robur,* apparently terrestrial, 10 September 2012, leg. G. Muñoz González, *GM 2555* (NBM-F-009322).

Western Asia. TURKEY: Karabük Province, Yenice district, around Yenice forest, on wood of fallen *Fagus orientalis*, 830 m a.s.l., 27 October 2012, leg. O. Kaygusuz (OKA-TR1445); Bursa Province, Uludag National Park, around Karabelen, on wood of *F. orientalis*, 705 m a.s.l., 25 October 2014, leg. O. Kaygusuz (OKA-TR1446); Bolu Province, Seven Lakes National Park, near Deringöl, on decayed wood of *F. orientalis*, 220 m a.s.l., 15 October 2015, leg. O. Kaygusuz (OKA-TR447); Aydın Province, Kuşadası district, around Güzelçamlı, on well-decayed branches of *Laurus nobilis*, 40 m a.s.l., 20 March 2016, leg. O. Kaygusuz (OKA-TR161); Kütahya Province, Domaniç district, on wood of *F. orientalis*, 950 m a.s.l., 5 October 2018, leg. O. Kaygusuz (OKA-TR162); Denizli Province, Pamukkale district, on wood of *Populus tremula*, 560 m a.s.l., 10 April 2019, leg. O. Kaygusuz (OKA-TRAB8); Isparta Province, Atabey district, on wood of *P. tremula*, 1120 m a.s.l., 8 June 2019, leg. O. Kaygusuz (OK-TR740).

**Notes**: The original description of *P. romellii* [10] is quite vague, and mainly characterizes this species as similar to *P. nanus,* with spores 6–7 μm, and growing on soil in Bavaria (Germany). Modern authors have interpreted *P. romellii* as a species in sect. *Celluloderma* with a brown pileus, a yellow stipe and no elongated elements in the pileipellis (e.g., [4,22]. This concept is accepted here, and the application of the name is stabilized by the selection of an epitype from central Europe (BRNM 761731) based on morphologically typical basidiomata fully corresponding with the description by Vellinga [4], and well-defined by nrITS + *TEF1-α* sequences, together with a lectotype based on original illustration [10]. Most authors have accepted the name *Pluteus lutescens* (Fr.) Bres. as a younger taxonomic synonym of *P. romellii* e.g., [4,22,23,26,54], and the same interpretation is accepted here.

The clade we accept as *Pluteus romellii* sensu stricto is recovered as well-supported in the analyses of the nrITS + *TEF1-α* dataset (Figure 1) and also in the individual nrITS (Figure 2) and *TEF1-α* (Figure 3) phylogenies.

*Pluteus pallescens* P.D. Orton was originally described as a species in sect. *Celluloderma* with a brown-colored, markedly hygrophanous pileus and a stipe “*white or whitish then discoloured dirty cream or pale dirty yellowish from base*” [20]. Orton emphasized the hygrophanous pileus and the broader pleurocystidia to separate *P. pallescens* from his concept of *P. nanus* (Pers.) P. Kumm., and the colors of the stipe (lacking grey colors) to separate *P. pallescens* from *P. griseopus* P.D. Orton. Later, after reexamining the original description and the type material of *P. satur* Kühner & Romagnesi, Orton concluded that both taxa were the same, and synonymized *P. pallescens* with *P. satur* [22]. It should be noted that, in the description of *P. satur* given by Orton [22], the stipe is described as “white or whitish then pale creamy grey” with no specific mention of the yellow base that was indicated in the original description of *P. pallescens*. Citérin & Eyssartier [26] reduced *P. pallescens* to a form of *P. satur*, characterized by a strongly striate and markedly hygrophanous pileus. We were able to obtain a nrITS sequence from the isotype collection of *P. pallescens*, and this sequence falls within the molecular variation of *P. romellii* s. stricto as accepted here (Figure 1 and Figure 2). We consider *P. pallescens* to be a younger synonym of *P. romellii*. Despite the suggestions of Orton [22] and Citérin & Eyssartier [26] of a close relation between *P. pallescens* and *P. satur*, our ongoing revision of sect. *Celluloderma* points in a different direction. The original material of *Pluteus satur* at the Conservatoire et Jardin botaniques de la Ville de Genève (G00052523 and G00052524) has differently shaped pleurocystidia, and moreover the nrITS sequences generated from this material indicates that *P. satur* belongs in the/cinereofuscus clade as defined by Menolli et al. [39] (Data not shown).

***Pluteus romellii* f. *albidus*** Ferisin, Riv. Micol. 4: 291 (2014).

Collection examined: ITALY: Farra d’Isonzo, Isonzo (Soca) river, 5 April 2014, leg. G. Ferisin (MCVE 28336, holotype); *ibidem*, 25 April 2018, leg. G. Ferisin (FG 25042018003).

**Notes**: This morphological variant of *P. romellii* is characterized by the white basidiomata [78]. Phylogenetic analyses of the type collection confirm that f. *albidus* is just an albino variant of the normally brightly colored *P. romellii* (Figure 4).

The existence of albino forms in several species of *Pluteus* sect. *Celluloderma* makes morphological identification challenging, as many species are defined by the colors of pileus and stipe. The relatively broad pleurocystidia of *P. romellii* f. *albidus* help in identifying this taxon in the absence of molecular data.

**Figure 6:**
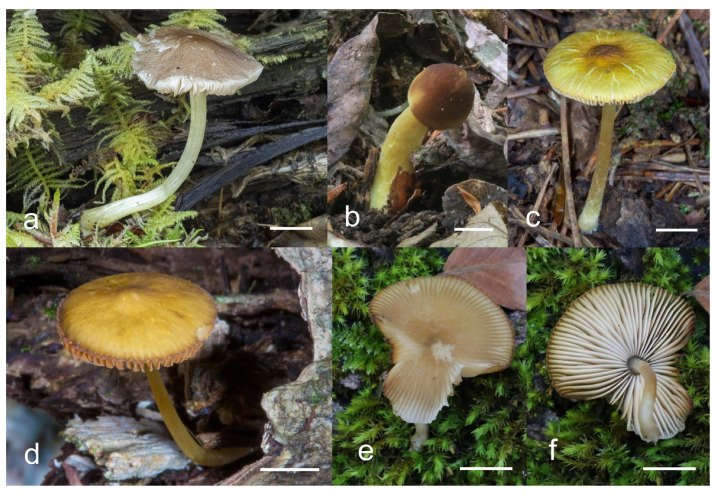
*Pluteus* aff. *romellii* basidiomata in situ: (**a**). LE 313599, (**b**). BRNM792987, (**c**). LE313340, (**d**). LE312975, (**e**,**f**). OKTR438. Scale bars 1 cm.

**Figure 7:**
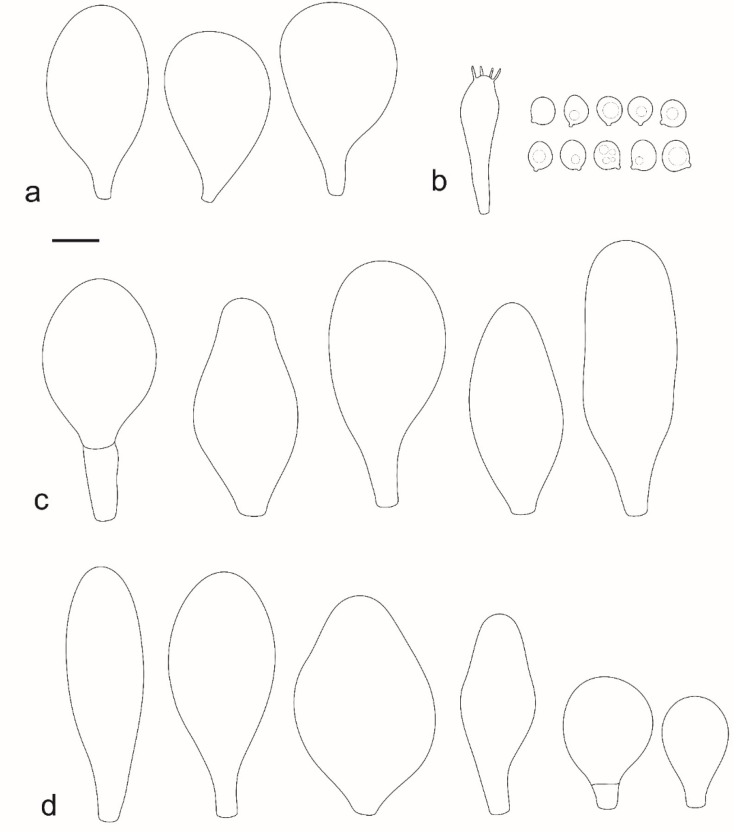
*Pluteus* aff. *romellii*: (**a**). pileipellis elements, (**b**). basidium and basidiospores, (**c**). pleurocystidia, (**d**). cheilocystidia. Scale bars 10 μm.

***Pluteus* aff. *romellii***Figure 6 and Figure 7.

Including: *Pluteus romellii* var. *luteoalbus* Ševčíková & Borov., in Krisai-Greilhuber et al., *Sydowia* 69: 245 (2017).

Holotype: CZECH REPUBLIC: Brno-venkov district, Kanice, Zadní Hády Nature reserve, *Querceto-Carpinetum*, on mossy fallen stem of unrecognized deciduous tree, 23 June 2016, leg. P. Ševčík, H. Ševčíková (BRNM 788199).

Syn.: *Pluteus romellii* f. *luteoalbus* (Ševčíková & Borov.) Lécuru, in Lécuru, Courtec. and P.-A. Moreau, Index Fungorum 384: 2 (2019).

Pileus 14–35 mm in diam., initially convex, then plano-convex to applanate; slightly hygrophanous or not, usually striate at the margin when mature; surface smooth, entirely wrinkled or wrinkled only at the center, sometimes cracked, pure yellow, yellow-brown, greyish brown or brown with yellow areas. Lamellae free, crowded to moderately crowded, ventricose, whitish when very young, later pink, with concolorous edges. Stipe 24–50 × 1.8–3.5 mm, cylindrical, solid, yellow when young or whitish at upper part and yellowish or pale yellow-brown beneath, longitudinally fibrillose. Smell indistinct, taste mild, indistinct.

Basidiospores [215, 9, 9] 4.9–8.7(–9.0) × 4.5–7.4, µm avl × avw = 5.7–7.3 × 5.1–6.5 μm, Q 1.01–1.28, avQ = 1.10–1.12; subglobose or globose, rarely ovoid or broadly ellipsoid, thick-walled. Basidia 25–31 × 6–8 µm, 4-spored. Pleurocystidia 39–76 × (14–)17–23(–28) µm, scattered, predominantly broadly utriform or broadly clavate, rarely cylindrical, some with mucous cap at apex, hyaline, thin- or slightly thick-walled. Cheilocystidia (20–)25–55(–70) × (10–)14–28(–37) µm, very numerous forming a sterile layer at the edge of lamellae, predominantly broadly clavate, rarely utriform or narrowly clavate, hyaline, thin- or slightly thick-walled. Pileipellis a hymeniderm, made up of spheropedunculate or broadly clavate elements, 28–50 × 14–27 µm, with brown intracellular pigment, thick-walled. Stipitipellis a cutis of cylindrical, hyaline, slightly thick-walled, 6–7 µm wide hyphae. Caulocystidia absent. Clamp connections absent in all studied tissues.

Habit, habitat, phenology and distribution: Often solitary, on decayed wood of deciduous trees (logs, fallen branches) including *Betula*, *Carpinus*, *Quercus*. May–January. Widely distributed in Eurasia, from Spain to the Russian Far-East, and possibly Japan.

**Additionalcollections examined:** Europe. CZECH REPUBLIC: Babice nad Svitavou near Brno, fallen trunk of *Carpinus*, 17 May 2017, leg. H. Ševčíková, P. Ševčík (BRNM792987). RUSSIA: Leningrad Region, vicinity of Lebedevka station, shore of Lebedinoye lake, birch forest with spruce in undergrowth, on rotten wood, 26 July 1998, leg. O.V. Morozova (LE 215032); Novgorod Region, vicinity of Valday, near Iversky Monastery, mixed forest (*Picea abies*, *Betula pendula*, *Populus tremula*, *Sorbus aucuparia*, *Alnus incana*), on rotten wood of deciduous tree, 20 August 2003, leg. O.V. Morozova (LE 217944); SPAIN. La Rioja, Navarrete, on wood, under *Quercus faginea*, 11 December 2015, leg. R. Martinez & L. Ballester, *LB 15121104* (NBM-F-009323); Sevilla, Las Navas de la Concepción, on *Quercus suber* wood, 29 November 2002, leg. N. Rodríguez-Ramos, *AJ 215* (NBM-F-009324).

Western Asia. TURKEY: Bolu Province, around Seven Lakes National Park, on wood of *Fagus orientalis*, 480 m a.s.l., 2 November 2015, leg. O. Kaygusuz (OKA-TR438); Denizli Province, Pamukkale district, on wood of *Populus tremula*, 530 m a.s.l., 10 March 2016, leg. O. Kaygusuz (OKA-TR21); Muğla Province, Fethiye district, under *Liquidambar orientalis*, 6 m a.s.l., 15 January 2018, leg. O. Kaygusuz (OKA-TR0820); Adana Province, Tarsus district, on fallen trunk of *Populus tremula*, 18 m a.s.l., 21 October 2020, leg. O. Kaygusuz (OKA-TR821).

Asia (incl. South Siberia and Far East). RUSSIA: Krasnoyarsk Territory, Sayano-Shushenskiy Biosphere Reserve, transect Karakem, mixed forest, on fallen trunk of *Betula pendula*, 21 August 2015, leg. A.A. Kiyashko (LE 303660); the same area, vicinities of Kurgol, mixed forest (*Betula pendula*, *Larix sibirica*), on fallen trunk of *Betula pendula*, 18 August 2015, leg. E.F. Malysheva (LE 313599); Primorye Territory, Land of the Leopard National Park, plateau near Ananjevka river, mixed forest (*Quercus mongolica*, *Carpinus cordata*, *Ulmus japonica*, *Abies holophylla*), on wood of deciduous tree, 1 September 2011, leg. A. Andreeva (LE 312975); Primorye Territory, Sikhote-Alin Biosphere Reserve, vicinity of Yasny field station, floodplain of Yasnaya river, forest with *Quercus mongolica* and *Pinus koraiensis*, on fallen branch of deciduous tree, 13 August 2012, leg. E.F. Malysheva (LE 313340); Primorye Territory, Land of the Leopard National Park, watershed between Ananjevka and Gryaznaya rivers, mixed forest (*Acer pseudosieboldianum*, *Quercus mongolica*, *Carpinus cordata*, *Abies holophylla*), on litter, 1 September 2011, leg. E.F. Malysheva (LE 313355). JAPAN: Hokkaido, Iwamizawa-shi, 11 September 2005, leg. S. Takehashi (TNS-F 12387).

**Notes**: The description above is based on the collections identified in Figure 1 as *Pluteus* aff. *romellii*. The holotype of *Pluteus romellii* var. *luteoalbus* falls within this group of sequences, but not all collections of *P.* aff. *romellii* fit the morphological concept of *P. romellii* var. *luteoalbus*. By its original description, *Pluteus romellii* var. *luteoalbus* differs from *Pluteus romellii* var. *romellii* by its completely different colors: a pileus that is yellow, darker at umbo; lamellae that are whitish to pale yellow (later pinkish by pink spore print), and a whitish stipe; context whitish in pileus, whitish to pale yellow in the stipe [79]. This variety was originally described and was only known from the Czech Republic. Lécuru [80] then recombined this taxon to form rank, as *Pluteus romellii* f. *luteoalbus* (Ševčíková & Borov.) Lécuru, but without any further taxonomic or molecular discussion.

*Pluteus splendidus* A. Pearson was originally described from England as a species with a bicolored pileus (chrome yellow at the center and yellow brown color towards the margin) and a chrome yellow or a lemon yellow stipe [61]. Vellinga & Schreurs [23] reexamined the original collections of *P. splendidus*, designated one of them as a lectotype, and concluded that *P. splendidus* was a synonym of *P. romellii*. Lécuru [80] recently recombined *Pluteus splendidus* A. Pearson as *P. romellii* f. *splendidus* (A. Pearson) Lécuru. We cannot currently define the identity of *P. splendidus*. Some collections of *P.* aff. *romellii* have a bicolored pileus, indicating that the name *P. splendidus* could be considered for this lineage. Unfortunately, no molecular data is available that reliably represents *P. splendidus*. Until molecular data become available from either the original collections of *P. splendidus* or modern collections from the UK, preferably from England, that fit the original description, the status of *P. splendidus* remains uncertain.

This clade is phylogenetically well-supported in the *TEF1-α* analysis (Figure 3), but receives no support in the nrITS (Figure 2) or combined analyses (Figure 1).

All available evidence points to *Pluteus* aff. *romellii* being a separate lineage from *P. romellii* s. stricto as accepted here, even if the nrITS phylogeny fails to recover *P.* aff. *romellii* as a distinct, well-supported clade. We refrain from formally describing this taxon as new, or from raising var. *luteoalbus* to species rank, as the name *P. splendidus* might be the correct name for this lineage. Should it prove in the future that the name *P. splendidus* is not applicable for this lineage, we recommend elevating taxon *Pluteus romellii* var. *luteoalbus* to species level as *Pluteus luteoalbus*.

***Pluteus fulvibadius*** Murrill, N. Amer. Fl. (New York) 10(2): 136 (1917) Figure 8 and Figure 9.

Holotype: USA: Oregon, Glen Brook, on the ground in woods, (in the original herbarium packet: “dense fir forest of mostly second growth, with few old oak trees at 400–1000 ft elevation”), 7 November 1911, leg. W.A. Murrill, *WA Murrill 760* (NY!). See Minnis & Sundberg [27] for a detailed type study.

Pileus 15–50 mm in diameter, hemispherical or campanulate when young, expanding to convex or plano-convex, often with a low, broad umbo; surface smooth, slightly to strongly rugose around the center, remaining intact or with small cracks in older specimens; with predominantly brown or yellow-brown colors (Munsell: 7.5YR 6/6–6/8, 5/4–5/8, 4/4–4/6; 10YR 6/6–6/8, 5/4–5/8, 4/4–4/6), sometimes with an olive-brown tint, young specimens might have predominantly green colors (5GY 3/4–5/4) that fade and become brown to olive-brown as they mature, darker at the center; dry, hygrophanous, slightly paler on drying; margin translucently striate. Lamellae crowded, free, ventricose, up to 8 mm broad, white or yellow when young, remaining yellow before turning pink as spores mature, with even or slightly flocculose edges, white or concolorous. Stipe 20–70 × 2–6 mm, cylindrical, with slightly broadened base (up to 8 mm wide); surface pale to bright yellow all over (2.5Y 8/6–8/8 or brighter), smooth or slightly fibrillose, sometimes with abundant white mycelium at base. Context in stipe and pileus white to pale yellow near the surfaces of pileus and stipe. Smell and taste indistinct.

Basidiospores [222, 10, 7] 5.5–7.6 × 5.0–7.0 μm, avl × avw = 6.4–7.2 × 5.6–6.1 μm, Q = 1.08–1.41, avQ = 1.13–1.29, broadly ellipsoid, more rarely ellipsoid or subglobose, some ovoid. Basidia 17–48 × 6–10 μm, 4-spored, clavate. Pleurocystidia 44–83 × 16–37 μm, mostly broadly clavate or utriform, some ovoid or obovoid; hyaline, thin-walled; common to scattered all over the lamellar faces; sometimes with a mucous cap covering the apex, with spores attached to it and trapped inside. Lamellar edge sterile. Cheilocystidia 33–61(–75) × 11–33 μm, narrowly clavate or narrowly utriform, rarely broadly clavate; hyaline, or very rarely some with a pale brown intracellular pigment, thin-walled, crowded, forming a well-developed strip. Pileipellis an epithelioid hymeniderm, with tightly packed individual elements: spheropedunculate, (broadly) clavate, pyriform, 30–61 × 16–42 µm; with evenly dissolved or aggregated intracellular brown pigment. Stipitipellis a cutis of cylindrical, slightly thick-walled, 6–10 µm wide hyphae; hyaline, or some with pale brown pigment. Caulocystidia absent or present, scattered, 30–55 × 17–30 μm, narrowly clavate or narrowly utriform, hyaline. Clamp connections absent in all studied tissues.

Habit, habitat, phenology and distribution: often gregarious, sometimes solitary, commonly growing on well-decayed wood of angiosperms, also recorded on mulch, on *Picea* litter, or apparently terrestrial. In temperate forests or transitional temperate/boreal forests. August–December. Western North America (Canada: British Columbia; USA: California, Colorado, Oregon); Eastern North America (Canada: Québec; USA: Michigan, Minnesota).

**Additional collections examined:** USA: California, Scotts Valley, Mission Springs Center, on decaying wood, 12 December 2012, leg. E.C. Vellinga, *AJ 815* (NBM-F-009325); Marin Co., Mount Tamalpais, Bolinas-Fairfax Road, near Alpine Lake, along roadside, 37.939062 -122.64209, in area with *Pseudotsuga menziesii*, *Notholithocarpus densiflorus*, *Sequoia sempervirens,* and *Arbutus menziesii*, 30 November 2011, leg. N. Nguyen *NN-120* (UC1861229); Santa Cruz, Big Basin SP, Santa Cruz, CA, 37.1665 -122.25157, 30 November 2013, on tanoak carcass, leg. C. Schwarz (UCSC-F-00818); Colorado, Weminuche Wilderness, Pine River Trail, in soil near *Picea* sp. litter, 18 August 2016, leg. D. Grootmyers, *Mushroom observer 270623* (NBM-F-009326); Michigan, Mackinac, Round Lake, NW of St. Ignace, 2 August 1962, on hardwood log in beech-maple-hemlock woods, leg. *R.L. Shaffer 3715* (MICH 69552). CANADA: Québec, Grondines, route Lefebvre, close to the railway, open area from cut-down mixed forest, on wood chips, 20 October 2021, leg. R. Lebeuf & A. Paul, *HRL3636* (NBM-F-009327); Sainte-Ursule, Chutes de Sainte-Ursule, 8 August 2021, hardwood forest of *Acer*, *Quercus* and *Fagus*, on wood chips, leg. R. Lebeuf, *HRL3391* (NBM-F-009328).

**Notes**: We accept *Pluteus fulvibadius*, originally described from Oregon (USA), as the correct name for one of the North American lineages in the *romellii* species complex. Based on the collections examined and publicly available records, this species is widely distributed in Western North America, and it is also present in Eastern North America, but it has only been recorded so far in the Northern states of the USA (Michigan, Minnesota) and in Canada (Québec). The morphological characteristics of our studied collections fit well the type collection revision by Minnis & Sundberg [27].

The nrITS sequences for the North American *P. fulvivadius* have 4–7 evolutionary events separating them from the Eurasian *P. romellii* and *P.* aff. *romellii*. Their *TEF1-α* sequences differ in 12–18 evolutionary events from *P. romellii* and *P.* aff. *romellii* (see Table S2 for a complete comparative overview). In all of the analyses, *P. fulvibadius* is recovered as a clade separate from the Eurasian collections, but with varying levels of support (Figure 1, Figure 2 and Figure 3).

We accept “*fulvibadius*” as the correct orthography for this species, despite Index Fungorum, MycoBank, MyCoPortal and other online sources accepting “*fulvobadius*” (at the time of writing the first version of this manuscript). Murrill uses “*fulvibadius*” in the original description, and consistently elsewhere in the original publication [13]. The reasoning behind the correction to “*fulvobadius*” is not clear to us. If Article 60.10 of the ICN [81] is followed, *fulvibadius* is the correct orthography, and no other grounds for orthographic correction listed under Article 60.1 apply here. After an informal discussion by one of the authors (A. Minnis) with the Nomenclature Committee for Fungi, some of these databases have corrected their spelling to “*fulvibadius*”.

Among the North American species in the *P. romellii* species complex, *P. fulvibadius* can be characterized by the relatively larger basidiomata, the lamellae often with bright yellow colors before the spores mature and the relatively larger basidiospores (see comparisons under *P. austrofulvus* and *P. parvisporus*).

*Pluteus vellingae* (see description below), while not part of the *romellii* species complex, is quite similar in morphology, and its geographical range overlaps with that of *P. fulvibadius*. *Pluteus vellingae* has generally smaller basidiomata, the lamellae are usually white or very pale yellow before sporulation and the basidiospores are slightly smaller (avl × avw = 5.7–6.2 × 5.1–5.6 μm), but molecular data might be necessary to confidently identify collections of both taxa.

Minnis & Sundberg [27] considered *Pluteus melleipes* Murrill a synonym of *P. fulvibadius*. Revision of the type material of *P. melleipes* revealed no significant differences with *P. fulvibadius* as described in Minnis & Sundberg [27], and as accepted here. Both Homola [21] and Minnis & Sundberg [27] suggested that there might be Eastern and Western North American variants under a broader concept of *P. fulvibadius* (as “*P. lutescens*” in Homola’s work) that accepted here. Minnis & Sundberg [27] suggested that if those variants were confirmed by future work the name *P. melleipes* (originally described from New York) could be adopted for the Eastern variant. No consistent differences, morphological or molecular, were found among the collections of *P. fulvibadius* from Western and Eastern North America studied here. The name *P. melleipes* was evaluated as a candidate for the species described here as *P. austrofulvus* and *P. parvisporus*, but morphology (including basidiospore size) and geographical distribution of *P. melleipes* fit *P. fulvibadius* better than any of the other North American taxa in the *romellii* species complex.

**Figure 8:**
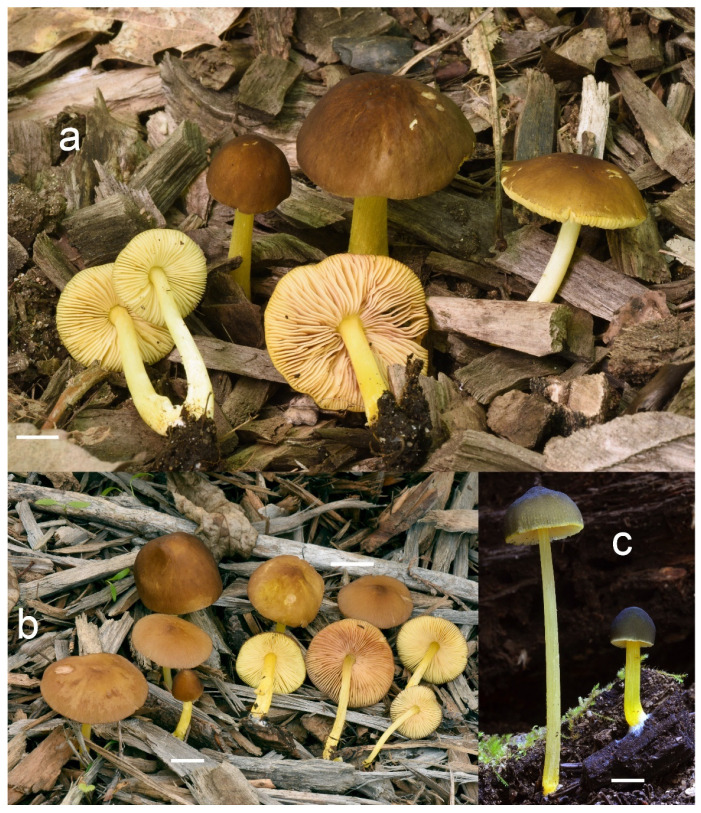
*Pluteus fulvibadius* basidiomata in situ: (**a**). HRL3391, (**b**). HRL3636, (**c**). UCSC-F-00818 (photo by Christian Schwarz). Scale bars 1 cm.

**Figure 9:**
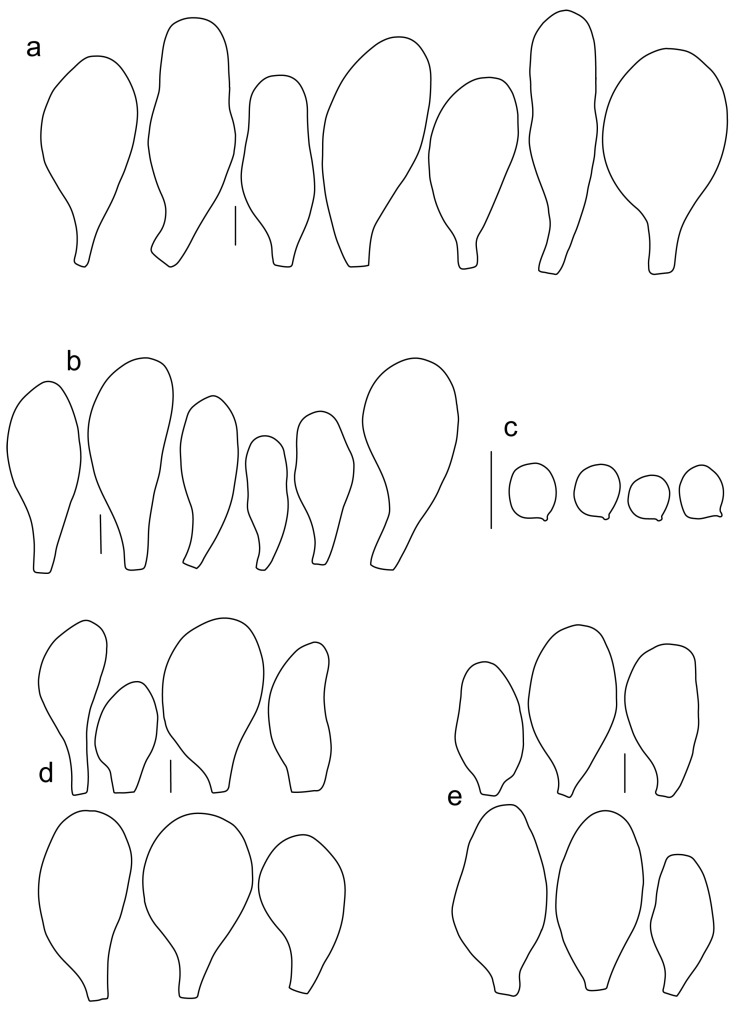
*Pluteus fulvibadius*: (**a**). pleurocystidia, (**b**). cheilocystidia (**c**). basidiospores, (**d**). pileipellis elements, (**e**). caulocystidia. Scale bars 10 μm.

One of the Californian collections studied here (UCSC-F-00818) has marked green colors in the pileus of young basidiomata (Figure 8), then fades to the usual brown colors. This characteristic led us to consider the name *Pluteus californicus* McClatchie [82]. This species was originally described from California as having a pileus with “*surface rugose-venose*, *hygrophanous*, *greenish drab*, *becoming cinnamon drab*”. It is unclear, however, whether *P. californicus* belongs to the/romellii clade. Murrill [13] and Smith and Stuntz [83] described this species as having yellow colors on the stipe, but that is not mentioned in the original description by McClatchie [82], who described the stipe as “*pale drab*”; moreover, neither of those authors studied any additional collections of *P. californicus*. No fresh material attributable to *P. californicus* has been collected since its original description. Green tones of the pileus are not uncommon in some of the taxa in the/cinereofuscus clade of sect. *Celluloderma*, and *P. californicus* could be considered for some the Western North American taxa in that group, but it might be best considered a doubtful name, since no modern collections examined by us can be confidently considered to correspond to *P. californicus*. 

*Pluteus rugosidiscus* Murrill is macroscopically similar to *P. fulvibadius*, especially to the specimens with green or olive tones in the pileus, but differs in the predominantly lageniform to fusiform pleurocystidia, with a long-to-short pedicel and/or long neck, and cheilocystidia predominantly lageniform with short neck or (broadly) utriform [2,29,53]. Phylogenetically, *P. rugosidiscus* is not closely related to any of the species in the/romellii clade [2].

**Figure 10:**
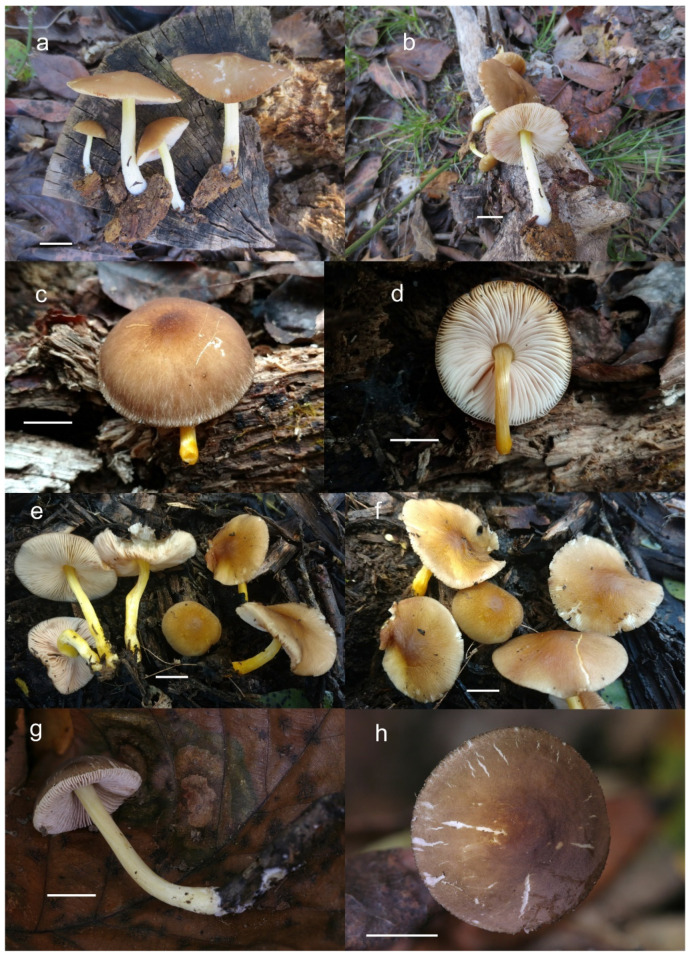
*Pluteus austrofulvus* basidiomata in situ: (**a**,**b**). AJ 857 (holotype), (**c**,**d**). iNaturalist 112016967, (**e**,**f**). iNaturalist 112219822, (**g**,**h**). iNaturalist 112280046. Scale bars 1 cm.

**Figure 11:**
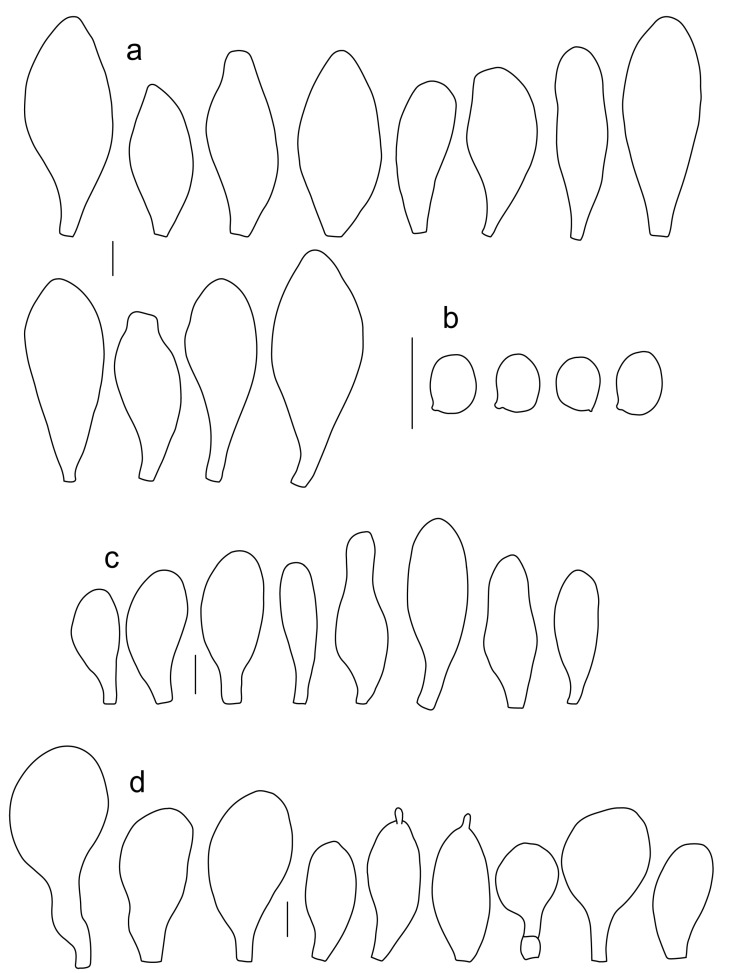
*Pluteus austrofulvus* (**a**). pleurocystidia, (**b**). basidiospores, (**c**). cheilocystidia, (**d**). pileipellis elements. Scale bars 10 μm.

***Pluteus austrofulvus*** Justo, Minnis, S. D. Russell & J. Kalichman, sp. nov. Figure 10 and Figure 11.

MB 844669.

Etymology: Makes reference to the yellow colors of the stipe, that characterize this species as part of the/romellii clade, and its more southern distribution relative to *P. fulvibadius.*

*Diagnosis*: Differs from *P. fulvibadius* by the smaller basidiospores and geographical distribution; differs from *P. parvisporus* by the bigger basidiospores, cracking pileus surface and gregarious habit.

Holotype: USA. Arkansas, Buffalo National River, Rush Landing, on decaying hardwood stump, 24 October 2013, leg. A. Justo, *AJ 857* (NBM-F-009329).

Pileus 10–35 mm in diameter, hemispherical or campanulate when young, expanding to convex or plano-convex, often with a low, broad umbo; surface smooth, slightly to strongly rugose around center or all over, often with cracks revealing the white context underneath; with predominantly brown colors (Munsell: 7.5YR 5/4–5/8, 4/4–4/6; 10YR 5/4–5/8, 4/4–4/6), sometimes olive-brown (2.5Y 6/6–6/8; 5/4–5/8) or yellow-brown (10YR 8/6–8/8, 7/6–7/8), darker at the center; dry, hygrophanous, may become very pale on drying; the margin translucently striate. Lamellae crowded, free, ventricose, up to 5 mm broad, white when young, remaining white before turning pink as spores mature, with even, serrulate or flocculose edges, white or concolorous. Stipe (7–)10–45 × 2–5 mm, cylindrical, with slightly broadened base; surface pale to bright yellow all over (2.5Y 8/6–8/8), smooth. Context in stipe and pileus white to pale yellow near the surfaces. Smell and taste indistinct.

Basidiospores [180, 8, 6] 5.0–7.2(–7.6) × 4.3–6.0(–6.5) μm, avl × avw = 6.1–6.4 × 4.9–5.2 μm, Q = (1.00–)1.08–1.30(–1.51), avQ = 1.17–1.27, subglobose to broadly ellipsoid, rarely globose or ellipsoid, some ovoid. Basidia 19–27 × 6–9 μm, 4-spored, clavate. Pleurocystidia 28–80(–86) × 16–36 μm, mostly ovoid, or broadly fusiform, also clavate or (narrowly) utriform; hyaline, thin-walled, rarely with some fine parietal incrustations; common to scattered all over lamellar faces. Lamellar edge sterile. Cheilocystidia 22–52(–63) × 8–19(–22) μm, (narrowly) clavate, (narrowly) utriform or ovoid; hyaline, thin-walled, crowded, forming a well-developed strip. Pileipellis an epithelioid hymeniderm, with tightly packed individual elements: spheropedunculate, (broadly) clavate, pyriform, a few shortly mucronate, 30–64(–69) × 14–42(–49) µm; with evenly dissolved or aggregated intracellular brown pigment. Stipitipellis a cutis of cylindrical, slightly thick-walled, 6–10 µm wide hyphae; hyaline, or some with pale brown pigment. Caulocystidia absent. Clamp connections absent in all studied tissues.

Habit, habitat, phenology and distribution: often gregarious, growing on well-decayed wood of angiosperms, often on fallen logs or stumps, more rarely in hardwood mulch. In temperate forests of Central, South Central and South Eastern United States. Recorded in areas with *Acer*, *Quercus* and *Carya* as dominant tree species. September–November. Eastern North America, USA (Arkansas, Georgia, Illinois, Indiana, Missouri, Tennessee).

**Additional collections examined:** USA: Arkansas, Buffalo National River, Buffalo Point, on cut bolt, 25 October 2013, leg. O. Miettinen, *AJ 864* (NBM-F-009330); *ibidem*, on uprooted tree, *AJ 860* (NBM-F-009331); Georgia, Clarke Co., Tallassee Highlands, on decaying hardwood, 17 November 2017, leg. J. Kalichman, *iNaturalist 112016967* (NBM-F-009332); Indiana, Westchester Township, 27 October 2019, leg. S. D. Russell, *iNaturalist 34997248* (NBM-F-009333); Illinois, Shelby Co., near Shelbyville, Hidden Springs State Forest, solitary on wood, 23 September 2006, leg. A.M. Minnis, *Minnis 6-09-23-3* (SIU); Missouri, Wayne Co., Mark Twain National Forest, Off State Highway 4, east of Williamsville, 24 October 1981, leg. D. Kost, *Sundberg X-24-1981-12* (ILL); Tennessee, Cumberland Co., Crossville, Hinch Mountain, 3 November 2018, on hardwood mulch, leg. J. Kalichman, *iNaturalist 112219822* (NBM-F-009334); Tennessee, Oak Ridge, Haw Ridge Park, 23 October 2020, on hardwood branch, leg. J. Kalichman, *iNaturalist 112280046* (NBM-F-009335).

**Notes**: *Pluteus austrofulvus* is recovered in the nrITS and *TEF1-α* phylogenies as a distinct lineage, separate from the other two North America taxa in the *romellii* species complex: *P. fulvibadius* and *P. parvisporus.*

*Pluteus fulvibadius* Murrill has a more northern distribution in Eastern North America (Minnesota, Michigan, and Québec). Macroscopically it has slightly bigger basidiomata, and the lamellae are bright yellow before turning pinkish as the spores mature. The basidiospores of *P. fulvibadius* are, on average, larger than those of *P. austrofulvus* (avl × avw = 6.4–7.2 × 5.6–6.1 μm in *P. fulvibadius*). *Pluteus fulvibadius* has predominantly broadly clavate or utriform pleurocystidia.

*Pluteus parvisporus* occurs sympatrically with *P. austrofulvus*, but both of their nrITS and *TEF1-α* sequences are quite different from each other (Table S2). *Pluteus parvisporus* tends to fruit solitary, has a non-cracking pileipellis, and can be separated microscopically by the smaller basidiospores with lower Q values (avl = 5.2–5.6 μm, avQ = 1.10–1.19) and the predominantly utriform or clavate pleurocystidia.

*Pluteus vellingae*, while morphologically similar, is phylogenetically distant from all taxa in the *romellii* species complex. Morphologically, it can appear similar to *P. austrofulvus*, but the pileus is not hygrophanous and it lacks an externally cracking surface. The pleurocystidia in *P. vellingae* are mostly clavate or broadly clavate, while in *P. austrofulvus* they are mostly ovoid or broadly fusiform.

**Figure 12:**
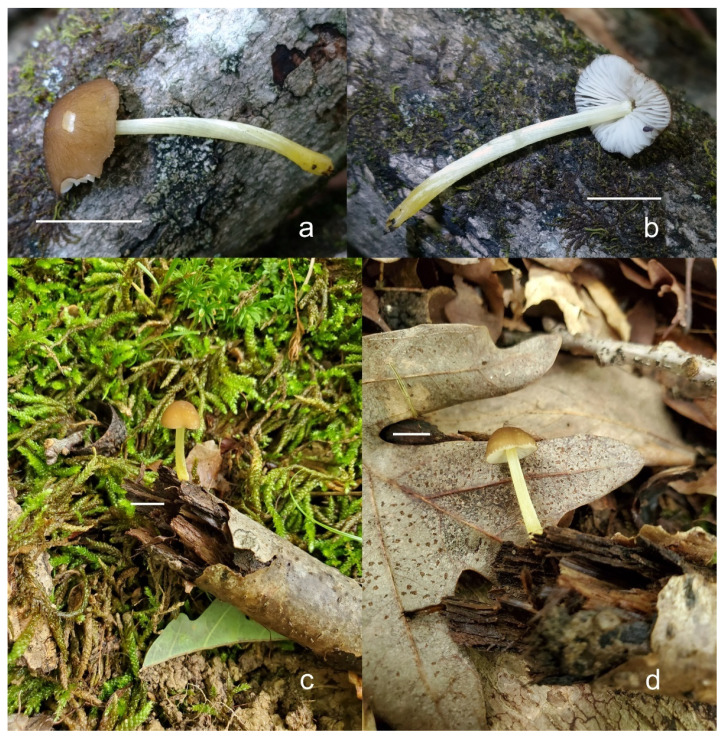
*Pluteus parvisporus* basidiomata in situ: (**a**,**b**). iNaturalist 112236342 (holotype), (**c**,**d**). iNaturalist 27586123. Scale bars 1 cm.

**Figure 13:**
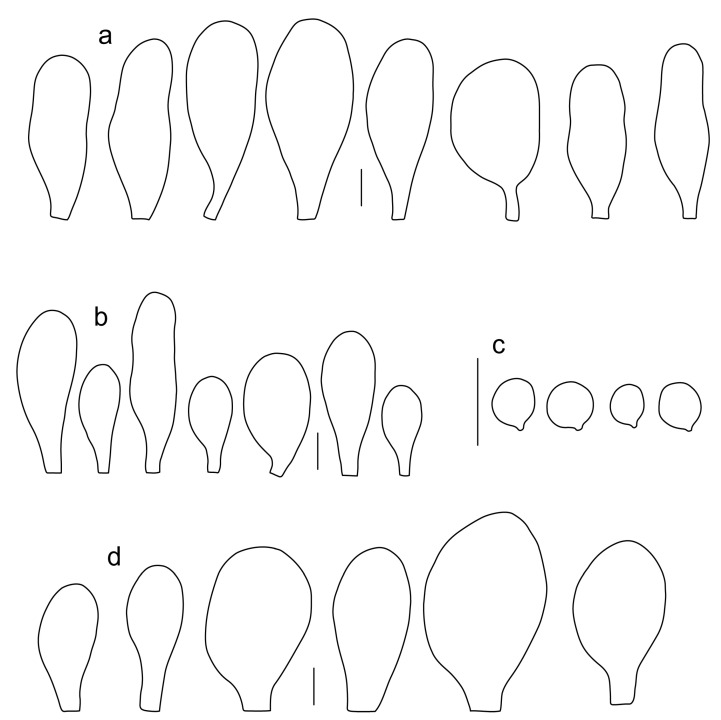
*Pluteus parvisporus* (**a**). pleurocystidia, (**b**). cheilocystidia (**c**). basidiospores, (**d**). pileipellis elements. Scale bars 10 μm.

***Pluteus parvisporus*** Justo, J. Kalichman & S. D. Russell, sp. nov. Figure 12 and Figure 13.

MB 844670.

Etymology: In reference to the relatively small basidiospores, that set apart this species from other members of the/romellii clade present in North America.

*Diagnosis*: Differs from *Pluteus fulvibadius* in its geographic distribution, smaller basidiomata, solitary habit and smaller basidiospores. Differs from *P. austrofulvus* and *P. vellingae* in the smaller basidiospores.

Holotype: USA: Tennessee, Oak Ridge, Haw Ridge Park, 21 June 2019, leg. J. Kalichman, *iNaturalist 112236342* (NBM-F-009336).

Pileus 10–15 mm in diameter, hemispherical or campanulate when young, expanding to convex or plano-convex, with or without a low, broad umbo; surface smooth, slightly to strongly rugose around center or all over, remaining entire, not cracking; with predominantly brown or yellow-brown colors (Munsell: 10YR 5/6–5/8, 6/6–6/8); dry, not markedly hygrophanous; margin slightly translucently striate. Lamellae crowded, free, ventricose, up to 3 mm broad, white when young, remaining white before turning pink as spores mature, with even or slightly flocculose edges, white or concolorous. Stipe 15–40 × 2–4 mm, cylindrical, with slightly broadened base; surface pale to bright yellow all over (2.5Y 8/6–8/8) or only at the base with the upper part white, smooth. Context white in pileus, in stipe white to pale yellow near the surface. Smell and taste indistinct.

Basidiospores [90, 3, 3] (4.5–) 4.8–6.5 × 4.0–5.5(–6.0) μm, avl × avw = 5.2–5.6 × 4.4–5.1 μm, Q = 1.00–1.26, avQ = 1.10–1.19, globose to broadly ellipsoid, some ovoid. Basidia 18–32 × 6–10 μm, 4-spored, clavate. Pleurocystidia 39–61 × 16–33 μm, mostly (narrowly) utriform or clavate; hyaline, thin-walled; common to scattered all over lamellar faces. Lamellar edge sterile. Cheilocystidia 19–55 × 8–20 μm, (narrowly) clavate, (narrowly) utriform, rarely broadly clavate; hyaline, very rarely some with pale brown intracellular pigment, thin-walled, crowded, forming a well-developed strip. Pileipellis an epithelioid hymeniderm, with tightly packed individual elements: spheropedunculate, (broadly) clavate, pyriform, 30–58 × 11–36(–44) µm; with evenly dissolved or aggregated intracellular brown pigment. Stipitipellis a cutis of cylindrical, slightly thick-walled, 6–10 µm wide hyphae; hyaline, or some with pale brown pigment. Caulocystidia absent. Clamp connections absent in all studied tissues.

Habit, habitat, phenology and distribution: solitary, growing on decayed wood of angiosperms, often on fallen logs or branches. In temperate forests of Central, South Central and South Eastern United States of America. Recorded in areas with *Acer*, *Quercus* and *Carya* as dominant species. June–October. North America, USA: Arkansas, Indiana, Tennessee.

**Additional collections examined:** USA: Arkansas, Ozarks National Forest, on decaying hardwood log, 23 October 2013, leg. A. Justo, *AJ 855* (NBM-F-009337); Indiana: Johnson Township, 24 June 2019, leg. S. D. Russell, *iNaturalist 27586123* (NBM-F-009338).

**Notes**: *Pluteus parvisporus* occurs in the same geographic area as *P. austrofulvus*, and both have been collected in the same or nearby localities, either at the same time or at different times in the season. *Pluteus parvisporus* tends to fruit solitary, and externally it lacks the cracking pileipellis that appears in many collections of *P. austrofulvus*. Basidiospore length and Q values are the best morphological characters to separate both taxa (see comments under *P. austrofulvus*).

The small basidiospores (with average length below 6 μm) separate *Pluteus parvisporus* from *Pluteus fulvibadius,* which, in addition, has a more northern distribution in Eastern North America (Minnesota, Michigan, and Québec). Macroscopically, it has bigger basidiomata, and the gills are bright yellow before turning pinkish as the spores mature.

*Pluteus vellingae*, while morphologically similar, is phylogenetically distant from all taxa in the *romellii* species complex. It differs from *P. parvisporus* in the larger basidiomata (pileus 7–40 mm), the pale yellow lamellae, the often gregarious fruiting, and the slightly larger basidiospores (avl × avw = 5.7–6.2 × 5.1–5.6 μm).

**Figure 14:**
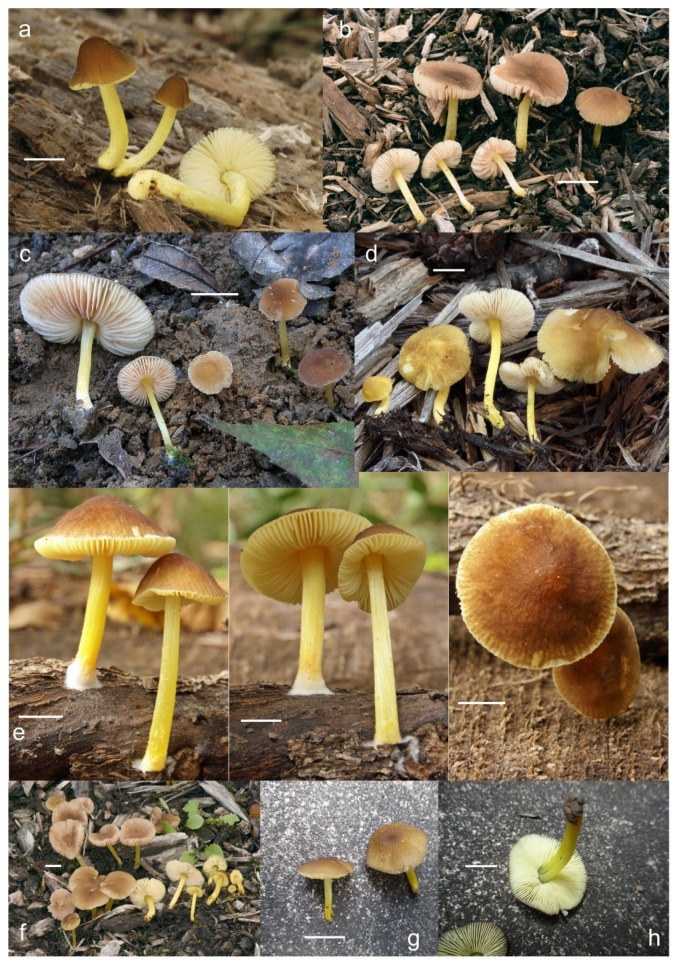
*Pluteus vellingae* basidiomata in situ: (**a**). HRL1462, (**b**). HRL3635, (**c**). GM 3260, (**d**). FG 23281, (**e**). FG 13772-17433, (**f**). HRL3646, (**g**,**h**). Mushroom Observer 281889 (photos by Dave Wasilewski). Scale bars 1 cm.

**Figure 15:**
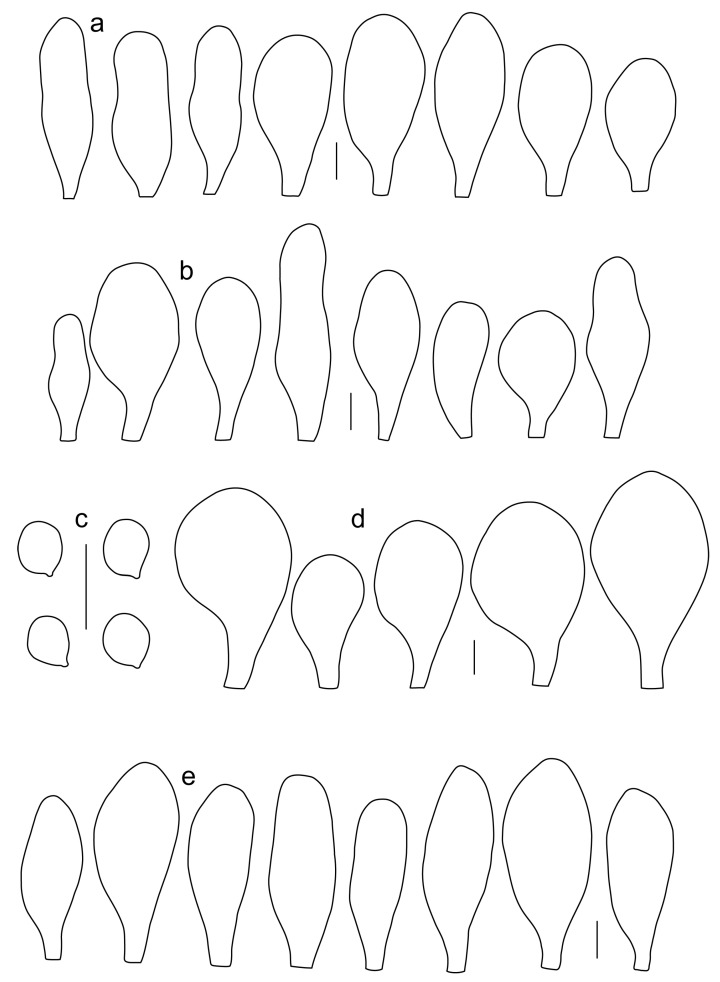
*Pluteus vellingae*: (**a**). pleurocystidia, (**b**). cheilocystidia (**c**). basidiospores, (**d**). pileipellis elements, (**e**). caulocystidia. Scale bars 10 μm.

***Pluteus vellingae*** Justo, Ferisin, Ševčíková, Kaygusuz, G. Muñoz, Lebeuf & S. D. Russell, sp. nov. Figure 14 and Figure 15.

MB 844671.

Etymology: In honor of mycologist Else C. Vellinga, collector of the holotype collection, in recognition of her exceptional contributions to mycology.

*Diagnosis*: Differs from *Pluteus fulvibadius* in the smaller basidiomata, with white or pale yellow gills before sporulation, and smaller basidiospores; differs from *P. austrofulvus* in the non-hygrophanous pileus and differently shaped pleurocystidia; differs from *P. parvisporus* in the larger basidiospores.

Holotype: USA: California, Alameda Co., Berkeley, Berkeley Marina (northern part), on woodchips, 28 February 2004, leg. E.C. Vellinga, *ECV 3201* (NBM-F-009339).

Pileus 7–40 mm broad, campanulate-convex or campanulate when young, expanding to convex or plano-convex or applanate; with or without a wide distinct umbo; not or slightly hygrophanous, translucently striate at the margin, rarely not, sometimes slightly eroded, surface smooth, dry; slightly to strongly rugose around center and from center towards margin; remaining intact or with small cracks in older specimens, brown to yellow-brown colors (Munsell: 7.5YR 8/6, 7/6–7/8, 6/6–6/8, 5/4–5/8, 4/4–4/6; 10YR 8/6–8/8, 7/6–7/8, 6/6–6/8, 5/4–5/8, 4/4–4/6), slightly paler at the margin. Lamellae free, crowded to moderately crowded, ventricose, 2–5 mm broad; cream or light yellow to yellow, later pinkish with or without yellow tinge, with even or slightly flocculose white or concolorous edges. Stipe 15–55 × 1–6 mm, cylindrical, often with slightly broadened base, smooth; surface pale to bright yellow (2.5Y 8/6–8/8 or brighter) or yellow with greenish tinge, longitudinally fibrillose, covered with concolorous floccules, with or without white tomentum at the base. Context white in the pileus, yellowish in the stipe. Smell indistinct, taste mild and indistinct.

Basidiospores [340, 12, 9] (4.7–)5.4–7.3(–7.7) × 4.3–6.3(–7.2) µm, avl × avw = 5.7–6.2 × 5.1–5.6 μm, Q = 1.00–1.26(–1.50), avQ = 1.05–1.19, globose, subglobose or broadly ellipsoid, rarely ellipsoid or ovoid, thick-walled. Basidia 16–36 × 6–11 µm, 4-spored, rarely 2-spored, mostly clavate. Pleurocystidia 30–55(–70) × 14–33(–40) µm, scarce to numerous, broadly clavate to clavate or ovoid, some utriform, hyaline, thin-walled. Lamellar edge sterile,. Cheilocystidia 34–76 × 11–46.5 µm, narrowly to broadly clavate or narrowly utriform, hyaline, thin-walled, forming a well-developed strip. Pileipellis an euhymeniderm with tightly packed individual elements, made of spheropedunculate, (broadly) clavate or pyriform elements, some mucronate, (17–)30–78(–82) × 17–46(–60) µm, with evenly dissolved or aggregated brown to yellow-brown intracellular pigment, thin-walled. Stipitipellis a cutis of slightly thick-walled, (4–)5–12 µm wide hyphae, hyaline or with pale yellowish content. Caulocystidia rarely present in the upper stipe or absent; (17–)30–78(–82) × 17–46(–60) µm, narrowly clavate, narrowly utriform or ovoid, colorless or with pale brown or yellow-brown content. Clamp connections absent in all studied tissues.

Habit, habitat phenology and distribution: gregarious, less frequently solitary, commonly growing on well-decayed wood of conifers or on conifer sawdust (*Abies*, *Picea, Pinus*), or on angiosperms (*Quercus*, *Fagus*, *Populus*), on woodchips, or apparently terrestrial. August to December in Europe, February in California, July to September in Eastern North America. Known from Europe (Czech Republic, Croatia, Slovenia/Italy border), Western Asia (Turkey), and North America (USA: California, Indiana, Pennsylvania; Canada: Québec, Ontario).

**Additional collections examined**: North America. CANADA: Québec: Pointe-Claire, boul. Saint-Jean, small city forest, on decomposed wood of *Quercus* or *Fagus*, 13 July 2013, leg. R. Lebeuf and A. Paul, *HRL1462* (NBM-F-009341); Grondines, route Lefebvre, close to the railway, open area from cut-down mixed forest, on wood chips, 20 October 2021, leg. R. Lebeuf and A. Paul, *HRL3635* (NBM-F-009342); *ibidem*, 24 October 2021, *HRL3646* (NBM-F-009343). USA: Pennsylvania, Sullivan Co., Loyalsock State Forest, apparently terrestrial along a dirt road, 9 July 2017, leg. D. Wasilewski, *Mushroom Observer 281889* (NBM-F-009340); Indiana: Pulaski, Winamac, 4 August 2018, *iNaturalist 15112653*, leg. S. D. Russell (PUL-F24687). 

Europe. CROATIA: Tor, Camping Lanterna, on *Pinus pinea* wood, 11 November 2017, leg. G. Ferisin (FG 13772-17433). CZECH REPUBLIC: Komňa, lom Rasová, coniferous wood near path, 22 August 2019, leg. V. Halasů, H. Ševčíková (BRNM 817769). SLOVENIA: Nova Goricȃ, Soča Park, on *Picea abies* wood, 7 October 2019, leg. G. Ferisin (FG 23281); *ibidem*, 14 October 2018, leg. G. Ferisin (FG 18030); Nova Goricȃ, Panovec Park, on *Picea abies* wood, 2 September 2019, leg. G. Ferisin (FG 02092019008). SPAIN: Navarra, Bertizarana, in the middle of a path, on muddy land, directly on soil, in mixed broadleaf forest (*Castanea* sp., *Corylus avellanea*) and abundant non-native plants (*Platanus* × *hispanica*, *Diospyros kaki*, bamboo, etc.), near a stream, 21 October 2018, leg. G. Muñoz González, *GM 3260*.

Asia. Western Asia. TURKEY: Isparta Province, near Gelincik district, on wood of *Populus alba*, 1150 m a.s.l., 30 November 2011, leg. O. Kaygusuz (OKA-TR512); Denizli Province, Acıpayam district, around Kelekçi town, in grass under *Populus tremula*, 800 m a.s.l., 12 December 2012, leg. O. Kaygusuz (OKA-TROKA1); *ibidem*, on wood of *Populus tremula*, 680 m a.s.l., 15 December 2013, leg. O. Kaygusuz (OKA-TRBA).

**Notes:***Pluteus vellingae* is characterized by a yellow-brown to brown, not or only slightly hygrophanous, mostly rugose pileus; scarce to numerous, broadly clavate to clavate or ovoid pleurocystidia; narrowly to broadly clavate or narrowly utriform cheilocystidia, globose to broadly ellipsoid basidiospores and growth on coniferous or deciduous wood. Molecularly, *P. vellingae* is quite different from all taxa in the *P. romellii* species complex and also from its two closest relatives in the/romellii clade, *P. parvicarpus* and *P. siccus* (Table S2). The separation of all these taxa based on morphological features could be challenging.

*Pluteus vellingae* has often (but not exclusively) been collected on coniferous wood, which is not a common habitat for species in the/romellii clade. Of the species described here, only *P. fulvibadius* has been confirmed to occur on coniferous wood or duff. *Pluteus fulvibadius* has generally larger basidiomata, with bright yellow lamellae that remain that color until sporulation, and larger basidiospores (on average 6.4–7.2 × 5.6–6.1 μm). *Pluteus austrofulvus* has a hygrophanous pileus, with an often-cracked surface, and predominantly ovoid to broadly fusiform pleurocystidia. *P. parvisporus* has smaller basidiospores (on average 5.2–5.6 × 4.4–5.1 μm).

*Pluteus romellii* has larger basidiospores (on average 6.5–7.3 × 5.3–6.1 μm), that are predominantly broadly ellipsoid (avQ = 1.16–1.24).

**Figure 16:**
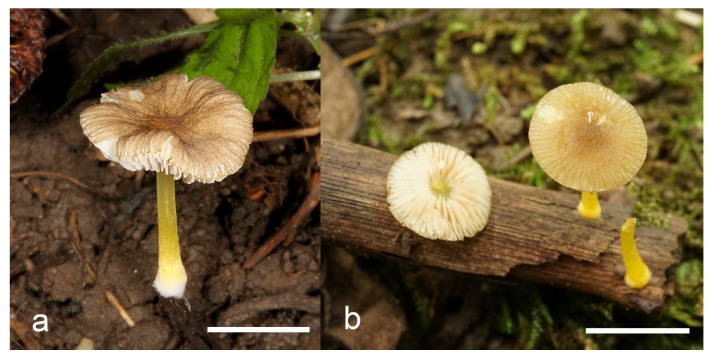
*Pluteus parvicarpus* basidiomata in situ: (**a**). LE 313357 (holotype), (**b**). LE 313631. Scale bars 1 cm.

**Figure 17:**
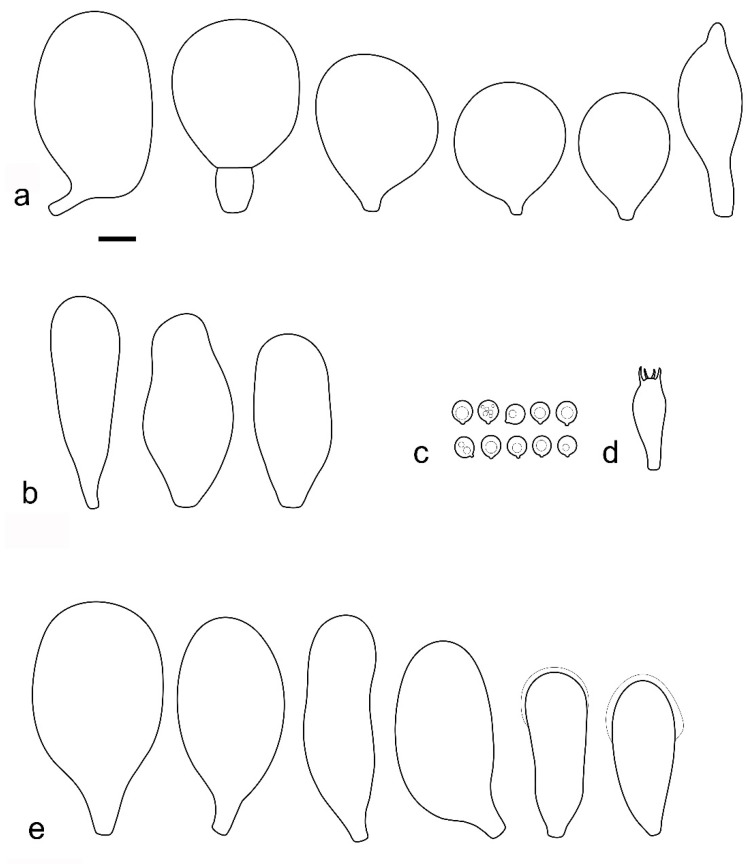
*Pluteus parvicarpus* holotype LE 313357: (**a**). pileipellis elements, (**b**). pleurocystidia, (**c**). basidiospores, (**d**). basidium, (**e**). cheilocystidia. Scale bar 10 μm.

***Pluteus parvicarpus*** E.F. Malysheva, sp. nov. Figure 16 and Figure 17.

MycoBank number: MB 844708.

Etymology: The epithet refers to the small size of the basidioma.

Diagnosis: Differs from *Pluteus vellingae* by less common and utriform pleurocystidia and smaller basidiospores, as well as distinct nrITS and *TEF1-α* sequences.

Holotype: RUSSIA: Far East, Primorye Territory, Ussuriysky Nature Reserve, vicinities of Peishula field station, floodplain *Ulmus*-forest, on fallen branches of deciduous tree in litter, 13 August 2011, leg. E.F. Malysheva (LE 313357).

Pileus 12–20 mm in diam., initially convex, then applanate to concave; not or slightly hygrophanous, sulcate-striate at margin; surface smooth and strongly venose at center, sometimes cracked at margin, ochre yellow (RAL 1024) or brown beige (RAL 1011), with an olive-brown (RAL 8008) center. Lamellae L = 36–48, free, rather distant, ventricose, 1.5–4 mm broad, pink, with concolorous edges. Stipe 12–15 × 1–2 mm, slightly broadened towards base, without or with small bulb, solid, bright lemon yellow (RAL 1012) or broom yellow (RAL 1032), pruinose, with white tomentum at base. Smell indistinct, taste not recorded.

Basidiospores [120, 4, 2] 4.5–6.0 × 4.2–5.5 µm, avl × avw = 4.9–5.2 × 4.6–4.9 μm, Q = 1.00–1.19, avQ = 1.07–1.08, globose, subglobose or very rarely ovoid, thick-walled. Basidia 23–27 × 7–8 µm, 4-spored, broadly clavate. *Pleurocystidia* 39–56 × 18–26 µm, very rare, utriform or broadly clavate, hyaline, thin- or slightly thick-walled. Cheilocystidia 22–63 × (9–)12–30 µm, numerous, forming a sterile layer at the edge of lamellae, predominantly broadly clavate or spheropedunculate, occasionally cylindrical or spathulate, many with mucous apical cap, hyaline, thin- or slightly thick-walled. Pileipellis a hymeniderm, made up of spheropedunculate, pyriform or broadly clavate elements, some with apical papillae, 24–52 × (15–)23–35 µm, with brown intracellular pigment, thick-walled. Stipitipellis a cutis of cylindrical, hyaline, slightly thick-walled, 5–7 µm wide hyphae. Caulocystidia absent. Clamp connections absent in all studied tissues.

Habit, habitat, phenology and distribution: solitary, on fallen branches of deciduous trees. With the current knowledge *P. parvicarpus* fruits in August and is known only from the Russian Far East.

**Additional collection examined**: RUSSIA: Far East, Primorye Territory, Ussuriysky Nature Reserve, on the road between Komarov’s house and Peishula field station, small-leaved forest, on a branch, 19 August 2020, leg. O.V. Morozova (LE 313631).

**Notes**: *Pluteus parvicarpus* is characterized by rather small or very small basidiomata with a yellowish brown pileus with olive-brown center, very rare pleurocystidia, cheilocystidia predominantly clavate, and globose or subglobose, relatively small basidiospores.

In the combined nrITS + *TEF1-α* phylogeny, *P. parvicarpus* occupies a sister position to *P. siccus*, from which it differs in 19 evolutionary events in its nrITS sequences and 29 in its *TEF1-α* sequences (Table S2). Morphologically, *P. parvicarpus* differs from *P. siccus* in the smaller basidiomata with brownish (not yellow) pileus, distinctly colored bright yellow stipe and geographical distribution in the Russian Far East.

*P. parvicarpus* differs from *P. vellingae* in the smaller basidiomata, less common and utriform pleurocystidia, smaller basidiospores and predominantly clavate/spheropedunculate cheilocystidia.

Among the species in the *P. romellii* species complex, only *P. parvisporus* has a similar basidiospore size. That species differs from *P. parvisporus* in the (narrowly) utriform or clavate cheilocystidia, and its distribution in Eastern North America. Molecularly, the nrITS and *TEF1-α* sequences of *P. parvisporus* and *P. parvicarpus* are very different from each other, with more than 50 individual differences in both cases (Table S2).

**Figure 18:**
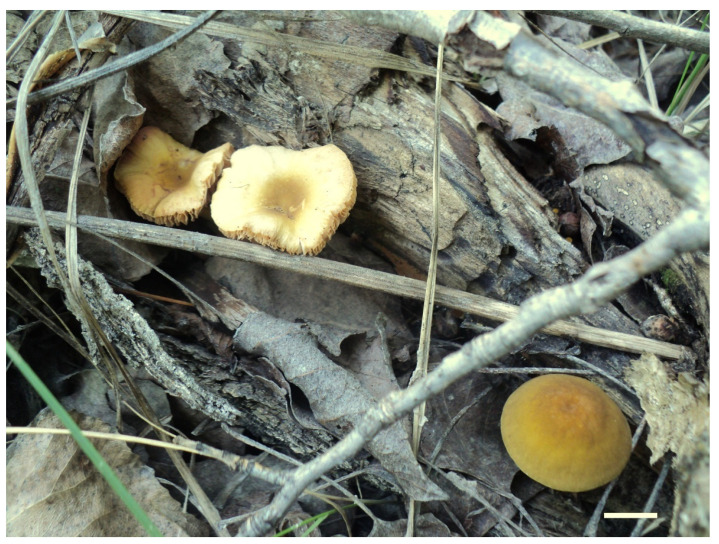
*Pluteus siccus* basidioma of holotype LE 313356 in situ. Scale bar 1 cm.

**Figure 19:**
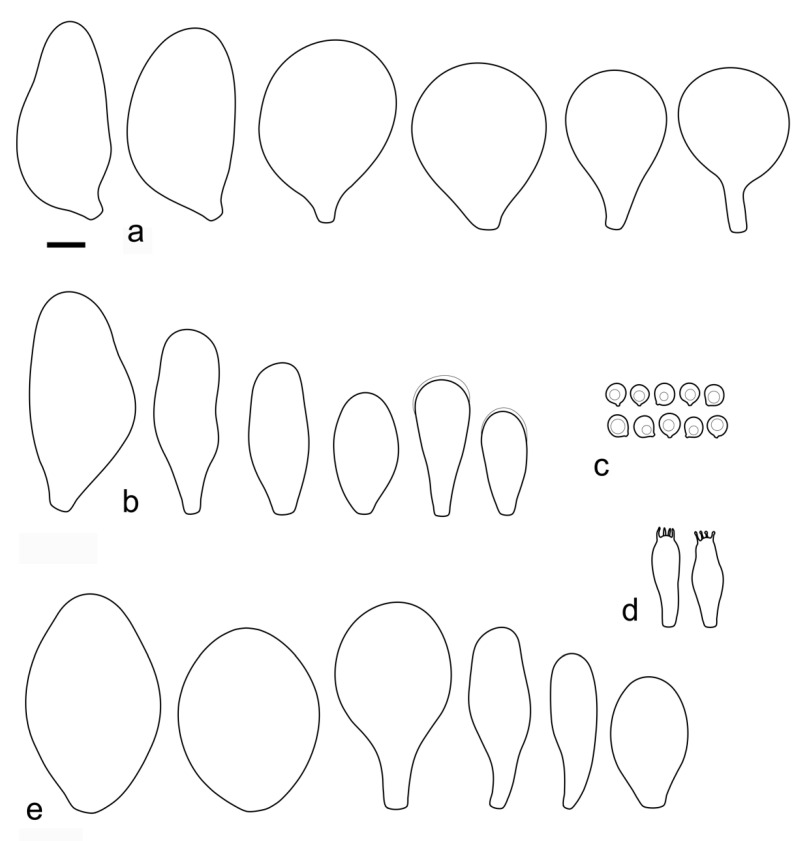
*Pluteus siccus*, holotype LE 313356: (**a**). pileipellis elements, (**b**). pleurocystidia, (**c**). basidiospores, (**d**). basidia, (**e**). cheilocystidia. Scale bar 10 μm.

***Pluteus siccus*** E.F. Malysheva, sp. nov. Figure 18 and Figure 19.

MycoBank number: MB 844712.

Etymology: The epithet refers to the peculiarities of the ecology of the species, its discovery in an arid area.

Diagnosis: Differs from *Pluteus romellii* by its yellow pileus with distinct green hue, smaller basidiospores and polymorphic cheilocystidia, as well as distinct nrITS and *TEF1-α* sequences.

Holotype: RUSSIA: European part, Volgograd Region, Kumylzhinsky District, Nizhnikhopersky nature monument, right bank of Khoper River, lowland forest, on decayed wood of deciduous tree, 18 July 2012, V.A. Dudka (LE 313356).

Pileus 15–30 mm in diam., initially convex, then applanate with concave center; slightly hygrophanous, sulcate at margin; surface smooth or slightly velvety, wrinkled at center, honey yellow (RAL 1005), ochre yellow (RAL 1024) or green beige (RAL 1000) with darker olive-brown (RAL 8008) center, when dry beige (RAL 1001). Lamellae L = 46–60, free, rather crowded, distinctly ventricose, 2–4 mm broad, pink, with concolorous or whitish flocculose edges. Stipe 25–35 × 1.5–2 mm, cylindrical or slightly broadened towards base but without bulb, solid, whitish at apex, sand yellow (RAL 1002) below, longitudinally fibrillose and flocculose at base. Smell indistinct, taste not recorded.

Basidiospores [90, 3, 1] 4.6–6.0 × (3.5–)4.5–5.5 µm, avl × avw = 5.0–5.3 × 4.7–4.8 μm, Q = 1.00–1.24(–1.43), avQ = 1.07–1.10, globose, subglobose or rarely broadly ellipsoid, thick-walled. Basidia 21–27 × 7–8 µm, 4-spored, broadly clavate. Pleurocystidia (25–)27–49(–58) × 9.5–18.5(–27) µm, scarce, utriform, broadly clavate or spathulate, some with mucous cap at apex, hyaline, thin- or slightly thick-walled. Cheilocystidia 33–55(–73) × (13–)17–37 µm, very numerous, forming a sterile layer at the edge of lamellae, rather polymorphic, predominantly broadly clavate or spheropedunculate, more rarely utriform or cylindrical, hyaline, thin- or slightly thick-walled. Pileipellis a hymeniderm, made up of spheropedunculate, broadly clavate or inflated-fusiform elements, 37–55 × 25–36 µm, with pale brown intracellular pigment, thick-walled. Stipitipellis a cutis of cylindrical, hyaline, slightly thick-walled, 6–8 µm wide hyphae. Caulocystidia absent. Clamp connections absent in all studied tissues.

Habit, habitat, phenology and distribution: in a small group, on decayed wood of deciduous tree. Known only from the holotype material collected in the Russian Far East.

**Notes**: *Pluteus siccus* is characterized by rather small basidiomata, a velvety pileus with greenish hue, utriform pleurocystidia often with slimy cap at apex, polymorphic cheilocystidia and small globose or subglobose basidiospores. *Pluteus siccus* is morphologically similar to *P. romellii* but differs from the latter in its velvety yellow pileus with a distinct green hue, smaller basidiospores and polymorphic cheilocystidia.

Based on molecular data, *P. siccus* occupies a very distant position apart from the *romellii* species complex, and phylogenetically it is more closely related to *P. parvicarpus* and *P. vellingae*. *Pluteus parvicarpus* significantly differs in smaller basidiomata with a brownish pileus and a bright yellow stipe, and geographical distribution in the Russian Far East. *Pluteus vellingae* differs by a brown rugose pileus, predominantly clavate pleurocystidia and larger basidiospores.

Among the species in the *romellii* species complex, only *P. parvisporus* has a similar basidiospore size, but that species has green hues on the pileus, fruits solitarily and occurs in Eastern North America.

**EXCLUDED AND UNCERTAIN TAXA**

***Pluteus sternbergii*** Velen. České Houby (Praze) 3: 610 (1921).

Holotype: CZECH REPUBLIC: Radotín, *Populus* stump, 18 August 1918 (herbarium Velenovský 173 (PRC), together with basidioma of “*P. dominii*“) (Velenovský [15: 610]).

Epitype (designated here): CZECH REPUBLIC: Mnichovice, in dumeto, August 1936, leg. J. Velenovský (PRM 154258, MBT10008040).

**Type study**: There are two basidiomata stored in one bottle with Velenovský’s solution [61]. One basidioma represents the holotype of *P. sternbergii*, while the second represents ‘*P. dominii*’, an unpublished name unknown to us. Both basidiomata have a lot of similar features described below, but different pleurocystidia (described separately).

Basidiospores (4.8–)5.0–7.0(–8.0) × 4.5––7.0 µm, Q = 1.00–1.20(–1.40), subglobose, globose or broadly ellipsoid, thick-walled. Basidia (18–)21–33(–37) × 7–11 µm, 4-spored. Pleurocystidia of one of the stored basidioma 40–50 × 15–25 µm, fusiform to broadly fusiform and wide obtuse apex with neck up to 10 µm, pleurocystidia of second basidioma 25–32 × 11–13 µm, narrowly utriform to clavate, thin-walled, hyaline. Lamellar edge sterile, some parts of lamellae also heterogenous or partly destroyed. Cheilocystidia (23–)30–47(–53) × (10–)12–40(–50) µm, mostly vesiculose to broadly clavate or clavate, rarely subfusiform or subutriform, thin-walled, hyaline. Pileipellis an euhymeniderm of spheropedunculate, broadly clavate and rare narrowly clavate to almost cylindrical elements (22–)25–44(–50) × (10–)12–28(–40) µm, colorless or with very pale brownish intracellular pigment, mostly thin-walled, rarely slightly thick-walled. Stipitipellis a cutis of 5–11 µm wide hyphae, hyaline to very pale yellow, thin to slightly thick-walled. Caulocystidia absent. Clamp connections absent in all studied tissues.

Habit, habitat and phenology (from the protologue): stump of *Populus* in valley. In temperate area of Central Europe, Czech Republic. May–July.

Notes: Velenovský characterized *Pluteus sternbergii* as having a brown, smooth and rugulose pileus, a smooth yellow stipe and whitish to pink lamellae [15] Based on the original description by Velenovský, Vellinga & Schreurs [23] placed *P. sternbergii* in synonymy with *P. romellii*.

The molecular analysis of the holotype deposited in PRC preserved in Velenovský’s solution repeatedly failed. Velenovský [15] mentioned globose spores, 7–8 µm, absence of pleurocystidia and obtusely rounded, vesiculose cheilocystidia. The holotype was stored together with another basidioma marked as “*P. dominii*”. Both basidiomata have rare pleurocystidia and similar pileipellis, cheilocystidia and spores, the latter probably mixed due to long joint storage. Pleurocystidia of one basidioma are bigger and mostly (broadly) fusiform (A), while smaller pleurocystidia of second basidioma are mostly narrowly utriform to clavate (B).

We were able to obtain a nrITS sequence from another collection (PRM 154258) identified by Velenovský as *P. sternbergii*. We include this sequence in our analyses (Figure 1 and Figure 2), but it is not closely related to any of the taxa in the/romellii clade. Broader analyses of sect. *Celluloderma* place *P. sternbergii* in the/cinereofuscus clade as defined by Menolli et al. [38] (data not shown). In view of the problematic status of the holotype of *P. sternbergii*, and to stabilize the usage of this name, we have selected collection PRM 154258 as the epitype of *P. sternbergii*.

***Pluteus splendidus*** A. Pearson, Trans. Br. mycol. Soc. 35(2): 110. 1952.

Syn. *Pluteus romellii* f. *splendidus* (A. Pearson) Lécuru, in Lécuru, Courtecuisse & Moreau, Index Fungorum 384: 2 (2019).

See comments under *Pluteus* aff. *romellii.*

***Pluteus melleipes*** Murrill N. Amer. Fl. (New York) 10(2): 129. 1917.

This species was originally described from New York (USA) by Murrill [13]. Different authors have interpreted this species differently in North American literature [21,83], but here we agree with Minnis & Sundberg [27] and consider this species a likely synonym of *Pluteus fulvibadius*. Before describing as new *P. austrofulvus*, *P. parvisporus* and *P. vellingae* we considered the possibility of using the name *P. melleipes* for each of these taxa. The final conclusion in all cases was that, of all the taxa in the *romellii* clade present in Eastern North America, (*P. fulvibadius*, *P. austrofulvus*, *P. parvisporus* and *P. vellingae*) it is actually *P. fulvibadius* the one that most closely matches the characteristics of *P. melleipes*, especially in basidiospore size and geographic distribution.

***Pluteus sulphureus*** Velen. České Houby (Praze) 3: 608. 1921.

Velenovský [15] characterized this species by a pale pileus covered with dark, appressed thin scales and a black-brown center, a smooth yellow stipe, lemon-yellow adnate lamellae, ovoid-globose spores measuring 4–5 µm and an absence of cystidia. Velenovský found it only once in a *Quercus* forest. Under the name *P. sulphureus*, there are three basidiomata preserved in the herbarium PRC (Velenovský no. 95!) in Velenovský’s solution, but none of them represent a *Pluteus* species. The adnate lamellae described in the protologue and our own observations of the type collection confirm that *P. sulphureus* does not represent a *Pluteus* species.

**Collection examined**: CZECH REPUBLIC: Roblín, *Quercetum*, July 191? (PRC, herbarium Velenovský 95).

***Pluteus chrysophaeus*** (Schaeff.) Quél., Mém. Soc. Émul. Montbéliard, Sér. 2 5: 82 (1872).

In the protologue of *Pluteus chrysophaeus*, the pileus was described as “saturate aureo”. However, the lectotype (Tom. III., Table CCLIII) established by Justo et al. [2] includes drawing of very young basidiomata with brown pilei which may also resemble basidiomata of the *P. romellii* group. *Pluteus chrysophaeus* has been interpreted in different ways by different authors [2,19,20,22,23,53] and is best considered a doubtful name without modern application.

## 4. Discussion

In the present paper, we circumscribe the limits of the commonly used name *Pluteus romellii*, based on morphological and molecular data, and formally lecotypify and epitypify that species in order to stabilize the usage of the name. We also provide a detailed description, and a phylogenetic delimitation, of *P. fulvibadius*, one of the North American species in the *romellii* species complex. Two additional North American species in the *romellii* species complex are described as new to science (*P. austrofulvus* and *P. parvisporus*). An additional Eurasian lineage in this species complex (*P.* aff. *romellii*) was detected in the phylogenetic analyses, but it is not formally described here, as more collections and molecular data are needed to further clarify its taxonomic and nomenclatural situation. Three other species which are morphologically similar to *P. romellii* were recovered in the phylogenies as part of the/romellii clade, but these taxa are not part of the *romellii* species complex as accepted here. They are newly described here as *P. vellingae*, *P. parvicarpus* and *P. siccus*.

Geographically, there are two groups of taxa: one Eurasian (*P. romellii*, *P.* aff. *romellii*, *P. siccus*, *P. parvicarpus*) and one North American (*P. fulvibadius*, *P. austrofulvus*, *P. parvisporus*). Only *P. vellingae* does not conform to this pattern and has a confirmed distribution on both sides of the Atlantic.

Generally, there are few unique morphological or ecological differences between the taxa described here. Nevertheless, we do interpret the phylogenetic lineages as separate species and therefore have to describe and name them. Without a correct understanding of the natural history of species in the *Pluteus romellii* complex, and a transparent taxonomy and nomenclature, it will be impossible to obtain more accurate data about the distribution, ecology, morphology and conservation status of these taxa. Regional endemics in this group need further studies to establish their possible conservation status. Morphological features are discussed below.

All Holarctic species with a brown pileus and yellow stipe in the/romellii clade have many common features and can be easily confused. Most species are characterized by a relatively small pileus in various shades of brown with a striate margin, yellow stipe, hyaline pleurocystidia, frequent thin-walled cheilocystidia and an euhymeniderm pileipellis.

Basidioma size varies from relatively small (pileus ≤ 15 mm) in *P. parvisporus*, *P. parvicarpus*, and *P. siccus* to medium-large (pileus ≥ 20 mm and up to 50 mm) in the other species described here. Extremely large basidiomata of *P. romellii*, with a pileus up to 80 mm, were collected from wood chips, which is a rich substrate often producing bigger basidiomata. On the other hand, basidiomata growing from small twigs tend to be very small. Pileus color is mostly brown to yellowish brown, but distinctly yellow pilei have been recorded in *P. siccus* and *P.* aff. *romellii* (incl. *P. romellii* var. *luteoalbus*); green or olive tones have been recorded in *P. fulvibadius*, *P. siccus* and *P. parvicarpus*.

*Pluteus austrofulvus* often has a pileus with cracks, revealing the white context underneath, a feature also observed in *P. parvicarpus,* while most other species usually have a non-cracked pileus. However, some collections can get this cracked surface in response to lack of humidity (e.g., *P. romellii* BRNM 825845). Lamellae of *P. romellii*, *P. vellingae*, and *P. fulvibadius* are often yellow, which might help for the first orientation in the field. While there is some intraspecific variability to this character, the North American collections of *P. fulvibadius* and *P. vellingae* seem to have specific patterns of lamellar coloration. In *P. fulvibadius*, the lamellae are often bright yellow, and the color remains intensely yellow until they turn pink as the spore mature. In *P. vellingae*, the lamellae start pale yellow, and their color often fades to almost white before the spores mature. The stipe of all species has some distinct yellow colors, either throughout the stipe length or just in its lower part. Some collections of *P. romellii* and *P.* aff. *romellii* have a white stipe, without distinct yellow colors.

Basidiospores of the species described here vary from globose to ellipsoid, but in most species, they are predominantly subglobose to broadly ellipsoid. Basidiospore size values have some overlap when comparing species, but the average values of length and width are useful for separating species. *Pluteus parvisporus*, *P. siccus* and *P. parvicarpus* have the smallest basidiospores (<6 µm long on average), while *P. romellii*, *P. fulvibadius* and *P. austrofulvus* have the larger ones (>6 µm long on average), while. *P. vellingae* and *P.* aff. *romellii* are somewhere in between. Pleurocystidia are very rare or scarce in *P. siccus* and *P. parvicarpus*, whereas they are scattered to common in the rest of the species described here. While there is a good degree of overlap in terms of morphology and size, some species tend to have a predominantly morphological characteristics that might be useful to identify collections; e.g., the pleurocystidia of *P. fulvibadius* are predominantly broadly clavate or utriform, while in *P. austrofulvus* they are mostly ovoid or broadly fusiform. A mucous cap was observed covering the apex of the pleurocystidia in *P. fulvibadius* (collection *HRL3636*) and *P. siccus* and the apex of the cheilocystidia in *P. parvicarpus*. All species have a well-developed strip of cheilocystidia covering the lamellar edge, often quite variable in shape and size, mostly hyaline but rarely with pale brown intracellular pigment. Caulocystidia are sometimes present in some taxa, but they are not a constant enough character to allow for species differentiation.

All species grow on deciduous wood, but *Pluteus fulvibadius* and *P. vellingae* are also known from coniferous wood. These species, as well as *P. romellii*, can apparently be terrestrial, but the presence of small pieces of decomposed wood in the soil can never be ruled out.

## 5. Conclusions

In this article, eight molecular lineages in the/romellii clade of the genus *Pluteus* are detected in the phylogenetic analyses, and their taxonomic status is discussed in detail. *Pluteus fulvibadius* and *P. sternbergii* are confirmed as separate species from *P. romellii*, while *P. pallescens* is synonymized with *P. romellii. Pluteus sulphureus* is rejected as not belonging to *Pluteus*. *Pluteus austrofulvus*, *P. parvicarpus*, *P. parvisporus*, *P. siccus* and *P. vellingae* are described as new species to science. *Pluteus* aff. *romellii* is recognized as a separate lineage from *P. romellii* sensu stricto, but further studies are necessary to clarify its taxonomic and nomenclatural status. A lectotype with an epitype collection for *P. romellii* and an epitype for *P. sternbergii* are designated here.

## Data Availability

Publicly available datasets were analyzed in this study. Those data can be found here: https://www.ncbi.nlm.nih.gov, accessed on 16 July 2022; https://www.mycobank.org, accessed on 16 July 2022.

## Supplementary Materials

The following supporting information can be downloaded at: https://www.mdpi.com/article/10.3390/jof8080773/s1. Table S1. Summary of Pluteus collections used in the phylogenetic analyses; Table S2. *Pluteus romellii* clade Differences in evolutionary events (nrITS above the shaded cells; TEF1-α below the shaded cells).
