# Supplementary material for: Holarctic Species in the Pluteus romellii Clade. Five New Species Described and Old Names Reassessed
Source: J Fungi (Basel). 2022 Jul 25;8(8):773. doi: 10.3390/jof8080773 (PMC9394350; doi:10.3390/jof8080773)
Supplement: Supplementary file 1 [file jof-08-00773-s001.zip › jof-1822570-supplementary.pdf]

Table S1. Summary of *Pluteus* collections used in the phylogenetic analyses. Sequences marked with an * were newly generated for this study.

Taxon	Collection	Country	nrITS	TEF1α
P. aff. *romellii*	AJ 215	Spain	HM562054	ON813269*
P. aff. *romellii*	BRNM 792987	Czech Republic	ON864083*	ON813272*
P. aff. *romellii*	LB 15121104	Spain	ON864082*	ON813271*
P. aff. *romellii*	LE 215032	Russia (Europe)	ON864086*	-
P. aff. *romellii*	LE 217944	Russia (Europe)	FJ774073	-
P. aff. *romellii*	LE 303660	Russia (Siberia)	KX216326	-
P. aff. *romellii*	LE 312975	Russia (Far East)	ON864081*	ON813270*
P. aff. *romellii*	LE 313340	Russia (Far East)	ON864084*	ON813273*
P. aff. *romellii*	LE 313355	Russia (Far East)	ON864085*	ON813275*
P. aff. *romellii*	LE 313599	Russia (Siberia)	ON864087*	ON813268*
P. aff. *romellii*	OKA-TR0820	Turkey	ON864088*	-
P. aff. *romellii*	OKA-TR21	Turkey	ON864090*	-
P. aff. *romellii*	OKA-TR438	Turkey	ON864091*	ON813274*
P. aff. *romellii*	OKA-TR821	Turkey	ON864089*	-
P. aff. *romellii*	TNS-F-12387	Japan	HM562123	-
P. aurantiorugosus	AJ 219	Spain	HM562041	-
P. aurantiorugosus	AJ 598	USA, New York	KM983697	-
P. aurantiorugosus	Bigelow 19232	USA, Massachusetts	HM562072	-
P. aurantiorugosus	Environmental sample	Estonia	UDB023616	-
P. aurantiorugosus	FG 10-2011	Italy	ON864102*	-
P. aurantiorugosus	GDGM41547	China	MK791275	-
P. aurantiorugosus	GM 2580	Spain	ON864101*	-
P. aurantiorugosus	GM 2692	Spain	ON864100*	-
P. aurantiorugosus	Hoseny 1740	USA, Michigan	HM562074	-
P. aurantiorugosus	ILLS 42433	USA, Illinois	HM562081	-

Taxon	Collection	Country	nrITS	TEF1α
P. aurantiorugosus	iNaturalist 30774897	USA, Indiana	ON006971	-
P. aurantiorugosus	LE 312803	Russia (Far East)	ON864105*	-
P. aurantiorugosus	LE 312815	Russia (Europe)	ON864103*	ON813296*
P. aurantiorugosus	LE 313555	Russia (Siberia)	ON864104*	-
P. aurantiorugosus	Mushroom Observer 110540	USA, Indiana	ON006945	-
P. aurantiorugosus	TNS-F 12391	Japan	HM562121	-
P. aurantiorugosus	Voucher 2847	Italy	JF908613	-
P. aurantiorugosus	Voucher 880	Italy	JF908608	-
P. aurantiorugosus var. *aurantiovelatus*	TO AVPP211	Italy	HQ654907	-
P. aurantiorugosus var. aurantiovelatus	TO AVPP212	Italy	HQ654908	-
P. aureovenatus	SP 393697	Brazil	FJ816663	KJ010056
P. aureovenatus	SP 394388	Brazil	HM562160	-
P. aureovenatus	SP 416735	Brazil	KM983702	-
P. austrofulvus	AJ 857	USA, Arkansas	KM983701	ON813290*
P. austrofulvus	AJ 860	USA, Arkansas	KM983699	ON813288*
P. austrofulvus	AJ 864	USA, Arkansas	KM983700	ON813289*
P. austrofulvus	iNaturalist 112016967	USA, Georgia	ON864095*	ON813291*
P. austrofulvus	iNaturalist 112219822	USA, Tennessee	ON864096*	ON813292*
P. austrofulvus	iNaturalist 112280046	USA, Tennessee	ON864097*	ON813293*
P. austrofulvus	iNaturalist 34997248	USA, Indiana	ON006963	-
P. austrofulvus	Minnis 6-09-23-3 (SIU)	USA, Illinois	HM562183	-

Taxon	Collection	Country	nrITS	TEF1α
P. austrofulvus	Sundberg X-24-1981-12 (ILL)	USA, Missouri	HM562105	-
P. castaneorugosus	LE 313071	Vietnam	MT611237	-
P. fulvibadius	AJ 815	USA, California	KM983698	ON813285*
P. fulvibadius	HRL3391	Canada, Québec	ON864094*	ON813287*
P. fulvibadius	JLF 4229	USA, Oregon	MK634598	-
P. fulvibadius	MCBS-167 (MIN)	USA, Minnesota	MIN055-09 (BOLD)	-
P. fulvibadius	MO 270623	USA, Colorado	ON864093*	ON813286*
P. fulvibadius	R.L. Shaffer 3715 (MICH)	USA, Michigan	HM562078	-
P. fulvibadius	UBC-F-33234	Canada, British Columbia	MG953973	-
P. fulvibadius	UC 1861229	USA, California	KC147675	-
P. fulvibadius	UCSC-F-00818	USA, California	ON864092*	-
P. globiger	ICN139025	Brazil	JQ065030	-
P. iguazuensis	NK I10	Brazil	KM983704	-
P. pallescens	K (M) 93678	UK, England	ON864073*	-
P. parvicarpus	LE 313357	Russia	ON864114*	ON813302*
P. parvicarpus	LE 313631	Russia	ON864115*	ON813303*
P. parvisporus	AJ 855	USA, Arkansas	ON864099*	ON813295*
P. parvisporus	iNaturalist 112236342	USA, Tennessee	ON864098*	ON813294*
P. parvisporus	iNaturalist 27586123	USA, Indiana	ON007017	-
P. paucicystidiatus	SP 394383	Brazil	HM562173	-
P. pauperculus	JAC11068	New Zealand	MN738636	-
P. pauperculus	JAC9790	New Zealand	MN738621	
P. phlebophorus	AJ81	Spain	HM562039	ON133554

Taxon	Collection	Country	nrITS	TEF1α
P. romellii	AJ 232	Spain	HM562062	ON813280*
P. romellii	BRNM 761731	Czech Republic	ON864065*	ON813278*
P. romellii	BRNM 816205	Czech Republic	ON864063*	ON813276*
P. romellii	BRNM 817530	Slovakia	ON864072*	ON813282*
P. romellii	BRNM 825844	Czech Republic	ON864066*	-
P. romellii	BRNM 825845	Slovakia	ON864070*	ON813281*
P. romellii	BRNM 825846	Czech Republic	ON864067*	ON813279*
P. romellii	Environmental Sample	Estonia	UDB015327	-
P. romellii	Environmental Sample	Estonia	UDB011747	-
P. romellii	FG 121020175	Italy	ON864069*	-
P. romellii	FG 1404201017	Italy	ON864068*	-
P. romellii	GM 2555	Spain	ON864062*	-
P. romellii	LE 262701	Russia (Caucasus)	ON864071*	-
P. romellii	OKA-TR1445	Turkey	ON864078*	-
P. romellii	OKA-TR1446	Turkey	ON864079*	-
P. romellii	OKA-TR161	Turkey	ON864076*	-
P. romellii	OKA-TR162	Turkey	ON864077*	-
P. romellii	OKA-TR447	Turkey	ON864080*	ON813283*
P. romellii	OKA-TRAB8	Turkey	ON864075*	-
P. romellii	OK-TR740	Turkey	ON864074*	ON813284*
P. romellii	PC 0714852	UK, England	ON864064*	ON813277*
P. romellii f. *albidus*	MCVE 28336	Italy	KM035790	-
P. romellii var. *luteoalbus*	BRNM 788199	Czech Republic	LT838190	-
P. rugosidiscus	BRNM761706	Slovakia	MH010876	LT991752
P. siccus	LE 313356	Russia	ON864113*	ON813301*

Taxon	Collection	Country	nrITS	TEF1α
P. stenotrichus	AJ 352	Dominican Republic	JN603201	-
P. sternbergii	PRM 154258	Czech Republic	ON864116*	-
P. sublaevigatus	SP 393694	Brazil	FJ816667	-
P. vellingae	BRNM 817769	Czech Republic	ON864108*	ON813297*
P. vellingae	ECV 3201	USA, California	AY854065	AY883433
P. vellingae	FG 07102019011	Slovenia	ON818995*	-
P. vellingae	FG 111120177	Croatia	ON818996*	-
P. vellingae	FG 14102018268	Slovenia	ON818997*	-
P. vellingae	FG02092019008	Slovenia	ON864112*	ON813299*
P. vellingae	GM 3260	Spain	ON864107*	ON813298*
P. vellingae	HRL1462	Canada, Quebec	ON864111*	-
P. vellingae	iNaturalist 15112653	USA, Indiana	ON006951	-
P. vellingae	iNaturalist 92133943	USA, Indiana	OM972345	-
P. vellingae	Mushroom Observer 281889	USA, Pennsylvania	ON864106*	-
P. vellingae	MycoMap 6997	USA, Indiana	MK564587	-
P. vellingae	OKA-TR512	Turkey	-	ON813300*
P. vellingae	OKA-TROKA1	Turkey	ON864110*	-
P. vellingae	OK-TRBA	Turkey	ON864109*	-
P. vellingae	TRTC 155719	Canada, Ontario	ONT270-08, (BOLD)	-
Pluteus sp.	AJ 842	Dominican Republic	KM983705	-
Pluteus sp.	SP 416739	Brazil	KM983703	-

Table S2

Pluteus romellii clade Differences in evolutionary events (nrITS above the shaded cells; *TEF1-α* below the shaded cells). Only evolutionary events shared by all sequences in the clade are counted and indels of multiple bases are counted as one single event.

	romellii	***aff. romellii***	***fulvibadius***	***austrofulvus***	***parvisporus***	***vellingae***	***siccus***	***parvicarpus***
romellii		4	7	15	15	>50	>50	>50
aff. romellii	10		4	8	10	>50	>50	>50
fulvibadius	12	18		7	12	>50	>50	>50
austrofulvus	7	12	15		17	>50	>50	>50
parvisporus	9	13	20	12		>50	>50	>50
vellingae	>50	>50	>50	>50	>50		24	27
siccus	>50	>50	>50	>50	>50	13		19
parvicarpus	>50	>50	>50	>50	>50	22	29	
